# Lentivirus-Mediated Trophoblast-Specific *Deptor* Knockdown Increases Transplacental System A and System L Amino Acid Transport and Fetal Growth in Mice

**DOI:** 10.1093/function/zqaf018

**Published:** 2025-03-25

**Authors:** Avery Kramer, Owen R Vaughan, Kenneth Barentsen, Johann Urschitz, Theresa L Powell, Thomas Jansson, Fredrick J Rosario

**Affiliations:** Department of Obstetrics and Gynecology, University of Colorado, Anschutz Medical Campus, Aurora, CO 80045, USA; EGA Institute for Women’s Health, University College London, London, WC1E 6HX, UK; Department of Obstetrics and Gynecology, University of Colorado, Anschutz Medical Campus, Aurora, CO 80045, USA; Institute for Biogenesis Research, University of Hawaii, Honolulu, HI 96822, USA; Department of Obstetrics and Gynecology, University of Colorado, Anschutz Medical Campus, Aurora, CO 80045, USA; Section of Neonatology, Department of Pediatrics, University of Colorado Anschutz Medical Campus, Aurora, CO 80045, USA; Department of Obstetrics and Gynecology, University of Colorado, Anschutz Medical Campus, Aurora, CO 80045, USA; Department of Obstetrics and Gynecology, University of Colorado, Anschutz Medical Campus, Aurora, CO 80045, USA

**Keywords:** placenta, maternal-fetal exchange, system A, system L, mechanistic target of rapamycin, fetal development, pregnancy complications

## Abstract

Mechanistic target of rapamycin (mTOR) signaling is a positive regulator of human placental function including system A/L amino acid transport activity. Placental mTOR signaling is inhibited in fetal growth restriction (FGR) and activated in fetal overgrowth in women; however, the causes of these changes in placental mTOR signaling are unknown. DEP (Dishevelled, Egl-10, Pleckstrin) domain containing mTOR-interacting protein (DEPTOR) is an endogenous inhibitor of mTOR. We tested the hypothesis that trophoblast-specific *Deptor* knockdown activates placental mTOR signaling and amino acid transport and causes fetal overgrowth. Using lentiviral transduction of blastocyst trophectoderm with a small hairpin RNA, we achieved 47% knockdown of placental *Deptor mRNA* expression, without altering fetal *Deptor mRNA* expression. Trophoblast-specific *Deptor* knockdown activated placental mTORC1 and mTORC2 signaling and increased trophoblast plasma membrane (TPM) LAT1 and SNAT2 protein abundance, and TPM system L and A transporter activity. In addition, *Deptor* knockdown increased in vivo transplacental system A and L amino acid transport and stimulated placental and fetal growth. In human FGR, placental DEPTOR protein expression was higher and negatively correlated with birth weight and microvillus plasma membrane system A activity. In conclusion, we provide mechanistic evidence that DEPTOR regulates placental mTOR signaling and amino acid transport and fetal growth in vivo. We speculate that modulation of placental DEPTOR is a promising target for intervention in pregnancies characterized by abnormal placental function and fetal growth.

## Graphical Abstract

**Graphical Abstract:**
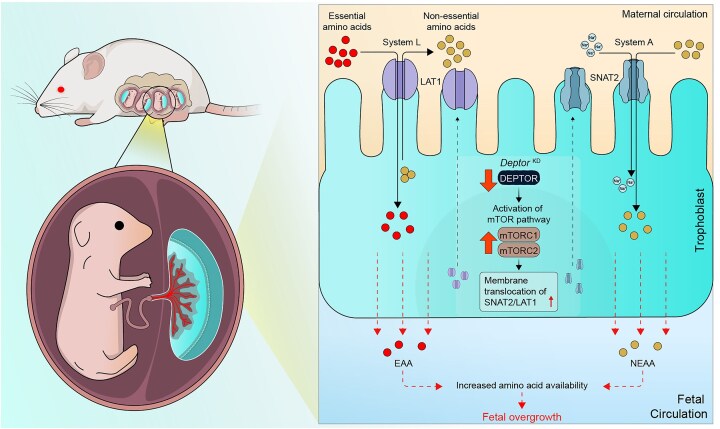

## Introduction

Abnormal fetal growth in utero significantly increases the risk of perinatal complications and predisposes the infant for type 2 diabetes mellitus, obesity, hypertension, dyslipidemia, and insulin resistance in later life.^1–4^ Fetal growth is highly dependent upon nutrient availability, which is dependent on placental nutrient transport. The activity of key placental nutrient transporters is decreased in fetal growth restriction (FGR)^5–8^ and upregulated in fetal overgrowth,^9–11^ suggesting that changes in the activity of key placental nutrient transporters may directly contribute to abnormal fetal growth.

Mechanistic target of rapamycin (mTOR) is a highly conserved serine threonine kinase that responds to nutrient availability and growth factor signaling to regulate cell growth and division, cell differentiation, tissue regeneration, and metabolism mediated by effects on gene transcription, protein translation, and post-translational modifications.^12^ mTOR resides in 2 distinct multiprotein signaling complexes referred to as mTORC1 and mTORC2.^13^ mTORC1 phosphorylates S6 kinase 1 (S6K1) and eukaryotic initiation factor 4E binding protein 1 (4E-BP1), which are involved in mRNA translation and protein synthesis.^13^ mTORC2 phosphorylates Akt, protein kinase C-α (PKC α), and serum and glucocorticoid-regulated kinase (SGK) and influences the actin skeleton and regulates cell metabolism.^14^ mTORC1 is a key nutrient sensor allowing cells and tissues to adapt their metabolism in response to nutritional cues.^12,14^ We and others have demonstrated that mTOR signaling is inhibited in human FGR^8,15–22^ and animal models of FGR.^17,23–38^ In contrast, activation of placental mTOR signaling is associated with fetal overgrowth.^10,19,39–43^

The amino acid transport system L is a major nutrient transport system responsible for Na^+^- independent transport of neutral amino acids (eg, leucine, isoleucine, and valine) across the plasma membrane by exchanging other neutral amino acids such as glutamine.^44^ System L activity is attributed to a large neutral amino acid transporter small subunit 1 (*Slc7a5*/LAT1) or 2 (*Slc7a8*/LAT2), which are covalently linked to the 4F2hc (*SLC3A2*) via a disulfide bridge.^44^ System A is a Na^+^-dependent transporter that actively transports small neutral amino acids. System A includes 3 distinct transporter isoforms, notably *Slc38a1*(SNAT1), *Slc38a2* (SNAT2), and *Slc38a4* (SNAT4), which have all been localized to the maternal-facing syncytiotrophoblast microvillous (MVM) plasma membrane of human placenta.^8^

DEPTOR, a protein containing 2 DEP (Dishevelled, Egl-10, Pleckstrin) domains, is a key endogenous inhibitor of both mTORC1 and mTORC2 complexes. DEPTOR is involved in multiple biological events, such as cell growth, apoptosis, autophagy, and cell differentiation.^45^ We demonstrated that *DEPTOR* silencing in primary human trophoblast (PHT) cells increased mTORC1 and C2 signaling.^46,47^ Furthermore, *DEPTOR* inhibition activated system A and L amino acid transport in PHT cells.^46,47^ However, compelling mechanistic evidence for the critical role of trophoblast DEPTOR in regulating placental function and fetal growth in vivo is lacking.

We hypothesized that trophoblast-specific *Deptor* knockdown activates placental mTOR signaling and amino acid transport and causes fetal overgrowth. We generated a lentiviral vector to deliver a small hairpin RNA (shRNA) *Deptor* gene knockdown construct to the blastocyst trophectoderm of embryonic day (E) 3.5 mouse embryos.^48–50^ At E18.5, we determined placental and fetal weight, in vitro system A and L amino acid transport capacity and transporter isoform protein expression in isolated trophoblast plasma membranes (TPMs), placental mTOR signaling, and in vivo placental amino acid transport capacity assessed by unidirectional maternal-fetal clearance of the tracer leucine and methyl-aminoisobutyric acid (MeAIB). Here, we report mechanistic evidence that DEPTOR regulates placental mTOR signaling and amino acid transport and fetal growth in vivo. We speculate that modulation of placental DEPTOR is a promising target for intervention in pregnancies characterized by abnormal placental function and fetal growth.

## Materials and Methods

### NSS and *Deptor* Lentiviral Vectors

Expression vector of mouse *Deptor* shRNA was constructed based upon the pLKO.1 vector^49,51^ and purchased from VectorBuilder Inc., Chicago, IL, USA (Vector ID: VB190821-1143qyf; Vector name: pLV[shRNA]-EGFP-U6 > m*Deptor*[shRNA#1]). The vector was constructed by inserting the following sequence into the plasmid backbone, which is present downstream of the human U6 small nuclear 1 promoter: targeting sequence, hairpin loop, and guide sequence. A human phosphoglycerate kinase 1 promoter-expressed enhanced green fluorescent protein (EGFP) open reading frame and an ampicillin resistance gene were also present in the plasmid backbone. A control lentivector pLV-EGFP-U6 > NSS (NSS), expressing an EGFP open reading frame and a nonsense targeting sequence (CCTAAGGTTAAGTCGCCCTCG), was purchased from Vector Builder (VectorBuilder Inc., Chicago, IL, USA). The NSS group was transduced with a lentiviral vector containing the EGFP reporter gene and a nonsense sequence (NSS) instead of the *Deptor shRNA* sequence. The NSS group serves a critical control group, showing that the observed changes in *Deptor* levels are not merely a consequence of the lentiviral transduction with a plasmid but specific to the lentiviral transduction with our *Deptor* knockdown plasmid. DNA was extracted using a commercial kit (Plasmid Maxi kit, Qiagen) after *Escherichia coli* glycerol stocks of *Deptor shRNA* and NSS plasmids were amplified in overnight cultures in LB (Lysogeny Broth) broth with 100 μg·mL^−1^ ampicillins. By cotransfecting 293FT cells with the isolated DNA (440 ng·mL^−1^, with Qiagen Polyfect 1% v/v in culture medium), along with packaging (330 ng·mL^−1^ pCMV delta 8.9) and envelope plasmids (140 ng.ml^−1^ pCMV-VSV-G), *Deptor* shRNA and NSS plasmids were subsequently packaged into vesicular stomatitis virus G–pseudotyped lentiviral particles. Packaging and envelope plasmids were obtained from the Functional Genomics Shared Resource at the University of Colorado Cancer Center. Thermo Fisher Scientific supplied the human 293FT (female embryonic kidney epithelial) cells, which were cultured at 37°C in complete media (Dulbecco’s modified Eagle’s medium containing 10% fetal bovine serum, 0.1 m m nonessential amino acids, 6 m m glutamine, 1 m m sodium pyruvate, and 1% pen-strep). Seventy-two hours after transfection, lentiviral particles were separated from the filtered culture media by ultracentrifugation of over 20% sucrose (81,166.8 g, 2 h), and they were then resuspended in PBS (Phosphate-buffered saline). By transducing 293FT cells with successive dilutions of the viral suspension and then utilizing flow cytometry to ascertain the proportion of EGFP fluorescent cells, the functional titer of each batch of lentivirus, expressed in transforming units per milliliter, was measured.

### Lentiviral Transduction of NSS and *Deptor* shRNA Constructs to Mouse Blastocysts and Surgical Embryo Transfer

**Figure 1.**
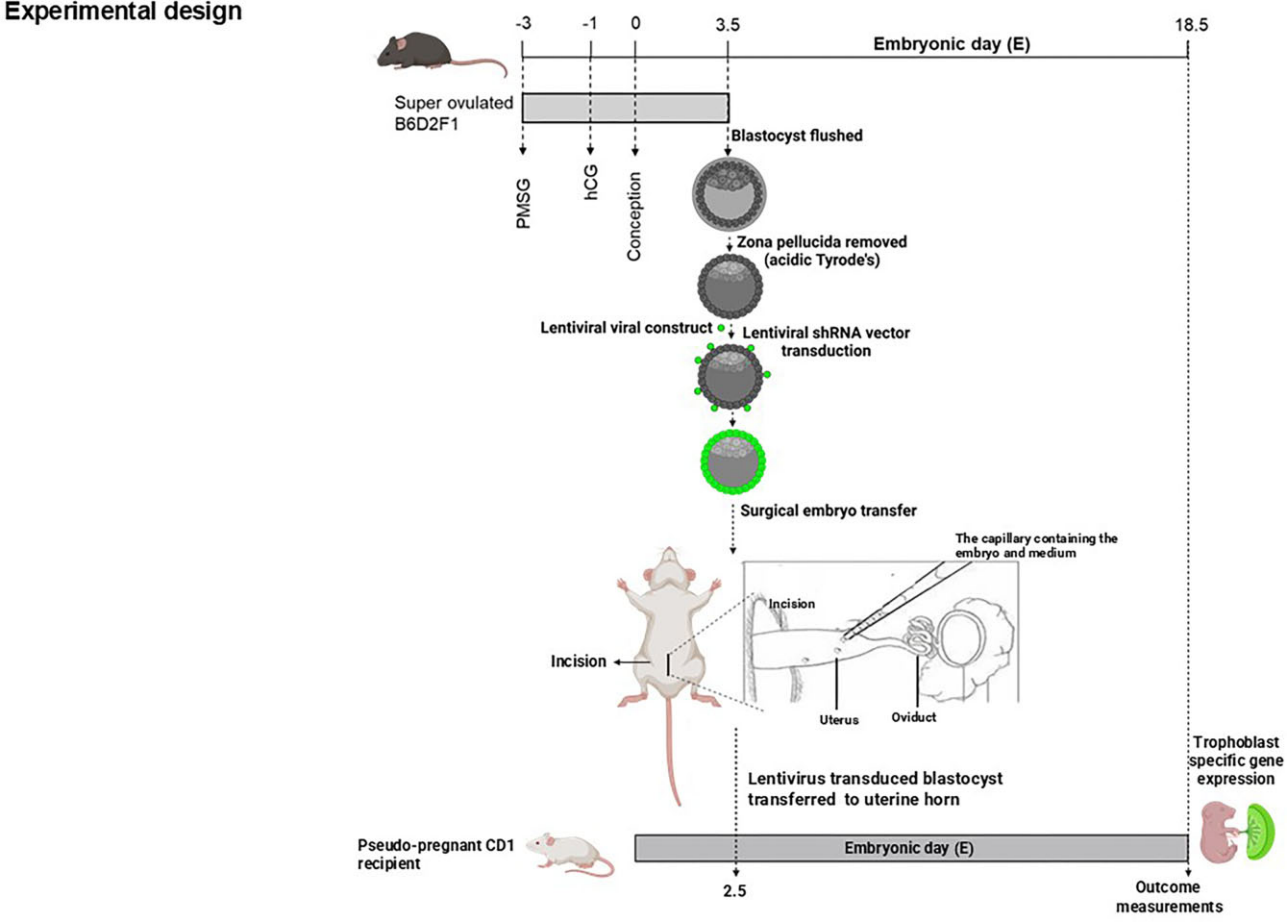
Experimental design. Schematic showing the experimental plan for lentiviral transduction of blastocysts. Blastocysts (B6D2F1) are transduced with lentiviral particles (shown in green) expressing control lentivector pLV-EGFP-U6 > NSS or pLV [shRNA]-EGFP-U6 > m *Deptor* [shRNA#1] constructs transferred to day 2.5 pseudopregnant (CD-1) mice for in vivo analyses.

All animal experimental protocols were performed at the University of Colorado Anschutz Medical Campus with approval from the Institutional Animal Care and Use Committee (Protocol #344). Charles River Laboratories (Wilmington, MA, USA) supplied the B6D2F1 mice, while CD-1 mice were purchased from Jackson Laboratories (Bar Harbor, ME, USA). All animals were housed in conventional light-dark circumstances, which were 14-h:10-h light:dark conditions, and they had ad libitum access to food and water. B6D2F1 was used as an embryo donor. The justification for this choice, B6D2F1 mice exhibits high reproductive performance, leading to improved fertilization rates and embryo viability compared to inbred strains, which make them reliable donors of embryos.^52^ In addition, B6D2F1 mice respond effectively to superovulation protocols, yielding a consistent and dependable number of oocytes, which is essential for efficient embryo collection. Using B6D2F1 as embryo donors is common due to their robust development and adaptability to various genetic modifications.^52^ To induce superovulation, female B6D2F1 mice (younger than 4 weeks, *n* = 30) were injected with human chorionic gonadotrophin (5 IU, intraperitoneally [ip], Sigma-Aldrich, St Louis, MO, USA) and pregnant mare serum gonadotrophin (5 IU, ip, Prospec, East Brunswick, NJ, USA). Hormone-treated B6D2F1 mice were then mated overnight with B6D2F1 males. The presence of a copulatory plug the following morning indicated that females successfully mated, which was designated as embryonic day (E) 0.5 (term = E19.5). Pregnant females were euthanized on E3.5 by cervical dislocation and CO_2_ asphyxiation. Their uteri were collected, and 5 mL of prewarmed M2 medium (M7167, Sigma-Aldrich, St Louis, MO, USA) were used to flush each horn. Flushed blastocysts were stripped of their zona pellucida by serial treatment in 3 drops of acidic Tyrode’s solution for approximately 10 s each. They were subsequently rinsed and incubated in embryo culture media (Embryo Max Advanced KSOM Embryo medium, MR-101-D, Millipore). Next, batches of 5–10 blastocysts were incubated with either 6.14 × 10^7^ (TU/mL) or 5 × 10^5^ TU/blastocyst transforming units of lentivirus in a total volume of 20 µL for 4 h in order to transduce the trophectoderm cells with either *Deptor* shRNA or NSS. After transduction, blastocysts were washed in 12 drops of embryo culture medium, and subsequently blastocysts were surgically transferred into pseudopregnant female CD-1 recipients (Figure 1). All in vitro procedures were carried out in low light conditions at 37°C.

In this study, we used CD-1 mice as an embryo recipient, which are an outbred strain known for their genetic variability. This variability reduces the risk of complications associated with inbreeding. Additionally, CD-1 mice have a higher rate of embryo implantation and larger litter sizes, making them ideal for embryo transfer.^53^ CD-1 embryo recipients were mated overnight with vasectomized B6D2F1 males to induce pseudopregnancy and subsequently underwent surgical embryo transfer 2.5 days post-copulation. Pseudopregnant CD-1 embryo recipients (*n* = 22) were administered preoperative analgesia (meloxicam, 1 mg/kg, ip) and then anesthetized with isoflurane (2%, inhaled). Under aseptic conditions and with the animal in sternal recumbency, a 0.5-cm incision was made in the flank skin above the right ovary, located 1 cm caudal to the rib cage. The ovary, oviduct, and distal uterine horn were extruded through the abdominal wall, and a 26-gauge needle was employed to perforate the uterus beneath the oviductal junction. Lentivirus-transduced blastocysts were subsequently delicately transferred into the uterine horn using a small volume of embryo culture media. The uterus and oviducts were subsequently reinserted into the body cavity, the body wall was closed with a single absorbable suture (5.0 vicryl), and the skin was secured with a 9-mm wound clip. The process was subsequently replicated for the left ovary. *Deptor shRNA* and NSS transduced blastocysts were transferred to contralateral horns of the same uterus and randomized to the left and right sides in each recipient. Postoperatively, recipient females recovered from anesthesia on a heated mat and then housed in pairs.

### Sex as a Biological Variable

One potential limitation of this study is that sex-specific effects of trophoblast-specific *Deptor* knockdown on placental function were not examined. Due to our experimental design, placentas from each litter were pooled to obtain sufficient material for isolation of TPMs, precluding the possibility of studying male and female placentas separately. However, the changes we report for most outcomes, including in mTOR signaling and TPM LAT1 and SNAT2 protein expression, were robust, compelling, and highly significant. If there were significant sexual dimorphism, the expected result would be either no effect of trophoblast-specific *Deptor* knockdown (female and males responding in opposite directions) or much more modest effects (one of the sexes not responding). Thus, we speculate that major sexual dimorphism in responses is unlikely.

### Collection of Placental Tissue and Fetus

Dams were euthanized at E18.5 by asphyxiation and cervical dislocation. Subsequently, fetuses and placentas were collected after laparotomy and quickly dried on blotting paper. Any residual adhering fetal membranes and decidua were removed from fetuses and placentas prior to weighing. NSS and *Deptor* shRNA placentas, respectively, from each litter (*n* = 8 litters) were pooled and washed briefly in PBS and transferred to ice-cold buffer D [250 m m sucrose, 10 m m HEPES (4-(2-hydroxyethyl)-1-piperazineethanesulfonic acid)-Tris, and 1 m m EDTA (Ethylene diamine tetra acetic acid) (pH 7.4) at 4°C] with protease and phosphatase inhibitor cocktail (Sigma-Aldrich, St Louis, MO, USA) added in a dilution of 1:1000. Pooled placentas were homogenized using a Polytron (Kinematica, Bohemia, NY, USA). The placental homogenate was frozen in liquid nitrogen and stored at −80°C until further processing and analysis. From each dam junctional zone (Jz) and labyrinth zone (Lz) from NSS and *Deptor* KD placentas were collected from placentas of each litter (*n* = 6 litters) and pooled and were flash frozen in liquid nitrogen and stored at –80°C until analysis.

### Analysis of Gene Expression

RNA was isolated from frozen placental labyrinth/junctional zone, fetal membrane, decidua, and other selected fetal tissues (liver, lung, heart, muscle, and brain). RNA was reverse transcribed utilizing commercial kits (RNeasy, Plus Mini kit, Qiacted gen, and High-Capacity cDNA RT kit, Invitrogen, Carlsbad, CA, USA). The expression of *Deptor* mRNA (forward sequence: 5′ AGCAGAGAGCTGGAACGC 3′; reverse sequence: 5′ CAGAGGCCTCCTTATGTTCA 3′) was quantified using SYBR Green qRT-PCR through the relative standard curve method, and reference to RNA28S expression.

### Isolation of Layer II TPM

TPM vesicles were isolated from pooled placental homogenates (*n* = 8 litters) using combined differential ultracentrifugation and Mg^2+^ precipitation, as previously described.^54^ Briefly, homogenates were subjected to serial centrifugation at 10 000 × *g* (10 min, 4°C), to remove tissue debris and nuclei, and supernatants were retained and centrifuged at 262, 5000 g (30 min, 4°C). The resultant membrane pellets were resuspended in buffer D and TPMs were precipitated by addition of MgCl_2_ (12 m m) and slow stirring, on ice. The precipitated membrane fraction was then washed and pelleted by ultracentrifugation (33 000 rpm, 30 min, 4°C), resuspended, and vesiculated using a Dounce homogenizer. Homogenate and TPM protein content were determined by the bicinchoninic acid assay (Thermo Scientific, Rockford, IL, USA) according to the manufacturer’s specifications and the enrichment of the preparation determined by the ratio of alkaline phosphatase activity, per unit protein in the TPM compared with homogenate as previously described. Average TPM enrichment ratios were similar between NSS (13.3 ± 2.0) and *Deptor* shRNA transduced (16.6 ± 3.6) placentas (*P* = .44).

### Immunoblotting

Placental homogenates and TPM samples from NSS and *Deptor* knockdown groups were processed for western blotting analyses as previously described.^55^ Isolated TPMs from both groups were used to assess the membrane protein expression of the system A amino acid transporter isoform SNAT 2 (*Slc38a2*) and the system L amino acid transporter isoform LAT1 (*Slc7a5*). A polyclonal SNAT2 antibody generated in rabbits was received as a generous gift from Dr V. Ganapathy and Dr P. Prasad at the University of Georgia, Augusta. Antibodies targeting the LAT1 were produced in rabbits and received as a generous gift from Dr Yoshikatsu Kanai, Osaka University, Osaka, Japan. The specificity of SNAT2 and LAT1 antibodies has previously been validated using gene-silencing techniques.^48,49^ In addition, mTOR signaling activity readouts in placental homogenates were measured by western blot for total and phosphorylated forms of S6 (Ser-235/236) and Akt (Ser-473). Target protein expression was normalized against total protein expression. Densitometry analysis of target protein bands of the immunoblots was performed with Gene Tools (SynGene). The mean density of the control (NSS) sample bands was given an arbitrary value of 1 for each protein target, and the data of the trophoblast-specific *Deptor* knockdown group are presented relative to the control.

### In Vivo Transplacental System A and System L–Mediated Transport

Transplacental amino acid transport capacity mediated by system L and A transport systems was assessed in a subset of E18.5 pregnant recipient females of both NSS and *Deptor* KD groups (*n* = 8 litters). Maternal-placental and maternal-fetal clearance was measured using radiolabeled ^3^H-leucine and ^14^C-methyl-aminoisobutyric acid (^14^C-MeAIB, the system A transporter–specific substrate). Dams were anesthetized with ketamine (60 mg/kg) and xylazine (6 mg/kg, both administered intraperitoneally) and positioned on a heated pad. The lateral tail vein was cannulated and flushed with heparinized saline with a 28-gauge needle connected to a 0.5-mL insulin syringe, through polyethylene (PE20) tubing (0.38 mm ID, about 12 cm in length, B.D. Intramedic 427405, Becton Dickinson, NJ, USA). A mixed bolus of ^14^C-MeAIB (50 µCi/kg) and ^3^H-leucine (250 µCi/kg) was administered by the tail vein, followed by flushing. At precisely 3 min following tracer injection, dams were euthanized with sodium pentobarbital (390 mg/mL, 100 µL, intravenous), and a cardiac blood sample was collected quickly in a heparinized syringe. The uterus was exposed through laparotomy, and fetuses and placentas were excised from the uterus and membranes, quickly blotted, and weighed. Subsequently, fetuses and placentas were minced and solubilized in Biosol (1 mL for placenta, 3 mL for fetus, National Diagnostics, Atlanta) overnight at 55°C. The maternal blood was subsequently centrifuged at 12 879 g for 4 min at 4°C, and the supernatant plasma was collected. Radioactivity in maternal plasma and in solubilized fetuses and placentas were determined by β-counting. The unidirectional maternofetal clearance [K_mf_, measured as microliters per minute per gram (μL⋅min^−1^⋅g^−1^)] was calculated as follows:

${{K}_{mf}} = \frac{{{{N}_x}}}{{AU{{C}_{0 - x}}\, \times \,W}}.$ where *N_x_* are counts (measured as disintegrations per minute) in the fetus taken at time *x* when the mother was killed. *AUC*_0−*x*_ is area under the maternal radiolabel concentration curve from time 0 to the time the mother was killed [measured as dpm per microliter (dpm⋅min⋅μL^−1^)]. *W* is the wet weight of the placenta (measured in grams).

### TPM System A and L Amino Acid Transporter Activity Measurements

We measured the system A and L amino acid transporter activity in isolated TPM from the NSS and *Deptor* shRNA transduced placentas using radiolabeled amino acids and rapid filtration techniques.^26,48^ TPM vesicles from NSS and *Deptor* KD groups were preloaded by incubation in ice-cold buffer containing 300 m m mannitol and 10 m m HEPES-Tris, overnight at 4°C. Afterward, TPM vesicles were pelleted by centrifugation and reconstituted in a minimal volume of mannitol buffer (final protein concentration: 5–10 mg mL^−1^). Membrane vesicle suspension was stored on ice until transport activity measurements were carried out. Immediately prior to transport activity measurements, TPM vesicles were warmed to 37°C. At time zero, 30 μL TPM vesicles were rapidly combined (1:2) with the incubation buffer containing [^14^C] methyl-aminoisobutyric acid (MeAIB, 150 μm, specific activity 58.7 mCi/mmol) with or without Na^+^ or L-[^3^H] leucine (0.375 μm, specific activity 60 000 mCi/mmol). Based on earlier time course studies,^26,48^ uptake at 15 s was used in all subsequent experiments. The uptake of radiolabeled substrate was stopped by adding 2 mL of ice-cold PBS. Following this, vesicles were rapidly removed from the substrate medium by filtration on mixed ester filters (0.45 μm pore size, Millipore Corporation, Bedford, MA, USA) and rinsed with 3 × 2 mL of ice-cold PBS. Each condition was assessed in duplicate for each membrane vesicle preparation in all uptake experiments. Filters were solubilized in 2 mL liquid scintillation fluid (Filter Count, PerkinElmer, Waltham, MA, USA) and counted. Relevant blanks were counted simultaneously and subtracted from counts and uptakes represented as pmol (mg protein)^−1^. Na^+^-dependent uptake of MeAIB, indicative of system A activity, was calculated by subtracting Na^+^-independent from total uptakes. The leucine-mediated uptake was calculated by subtracting nonmediated transport, as assessed in the presence of 20 m m unlabeled leucine, from the total uptake.

### Placental DEPTOR Protein Expression in Human AGA and FGR Samples

With approval from the University of Western Ontario’s Health Sciences Research Ethics Board (HSREB) and written informed consent from participants, pregnant women were recruited at St Joseph’s Hospital in London, ON, Canada. Exclusion criteria included pregnancies complicated by congenital infections, chromosomal or congenital anomalies, diabetes, thyroid disorders, chronic hypertensive disorders, or preeclampsia. Gestational age was determined based on the last menstrual period and was confirmed by ultrasound in the first trimester. Clinical information was abstracted from antenatal records, which included standard tests for evaluating fetal health and assessing risk factors as part of routine surveillance. The diagnosis for the FGR group was based on the estimated fetal weight in the last trimester, which was determined by fetal ultrasound biometry and calculated using the Hadlock II formula.^56^ Birth weight percentiles were calculated according to gender and gestational age, using standardized growth charts.^57,58^ The appropriate-for-gestational age (AGA, control) group consisted of normal pregnancies without medical or obstetric complications except preterm birth in the majority of cases. The placentas from the control group were obtained from preterm deliveries (*n* = 12, gestational age range 27–36 weeks) and from uncomplicated term pregnancies (*n* = 7) where the birth weight was between the 25th and 75th percentiles for the corresponding gestational age. FGR was defined as a birth weight less than the third percentile for gestational age. Placental insufficiency was determined by an abnormal umbilical artery Doppler assessment.^59^ The placentas from the FGR group were obtained from both preterm (*n* = 14, gestational age range 27.9–36.4 weeks) and term deliveries (*n* = 11).

Placentas from pregnancies complicated by FGR and women delivering appropriate-for-gestational age (AGA) infants were collected within 15 min of delivery as described elsewhere.^8^ Briefly, the decidua basalis and the chorionic plate were removed, and the villous tissue was dissected and rinsed in cold physiological saline. The villous tissue was transferred to cold buffer D (250 m m sucrose, 10 m m HEPES, and pH 7.4) containing 1:100 dilution of protease and phosphatase inhibitors (Sigma-Aldrich, St Louis, MO, USA) and homogenized on ice with a Polytron (Kinematica, Luzern, Switzerland). The placental homogenates were frozen in liquid nitrogen and stored at −80°C until further processing. The expression of DEPTOR was determined in placental homogenates using western blot as described earlier. Placental MVM system A activity for AGA and FGR samples used in the current study has been reported previously.^8^ It was reported that MVM system A activity in the FGR group was 72% lower as compared with the AGA group.^8^ In this study, we also analyzed the relationship between the placental DEPTOR expression and MVM system A activity and birth weight in human AGA and FGR samples.

### Data Presentation and Statistics

GraphPad Prism 10.4.0 software was used to analyze the data. Data are presented as mean ± SEM. All placentas in a litter of the respective genotypes (NSS and *Deptor* KD) were pooled prior to the molecular analysis and transport assays. Thus, *n* represents the number of litters. The distribution of data was assessed by the D’Agostino & Pearson and Shapiro-Wilk tests. When data showed normal distribution (NSS and *Deptor* KD), statistical significance between NSS and *Deptor* KD was determined by paired Student’s *t*-test (*P* < .05). When data were not normally distributed, the Wilcoxon matched-pairs signed rank test (non-parametric) was used. The frequency distribution of individual fetal weights in NSS and *Deptor* KD groups was determined by Gaussian curves fitted with least-squares nonlinear regressions and compared using an extra sum-of-squares *F*-test.

## Results

### Placental DEPTOR Protein Expression Is Inversely Correlated With Birth Weight and MVM System A Amino Acid Transport Activity in Human FGR

**Figure 2.**
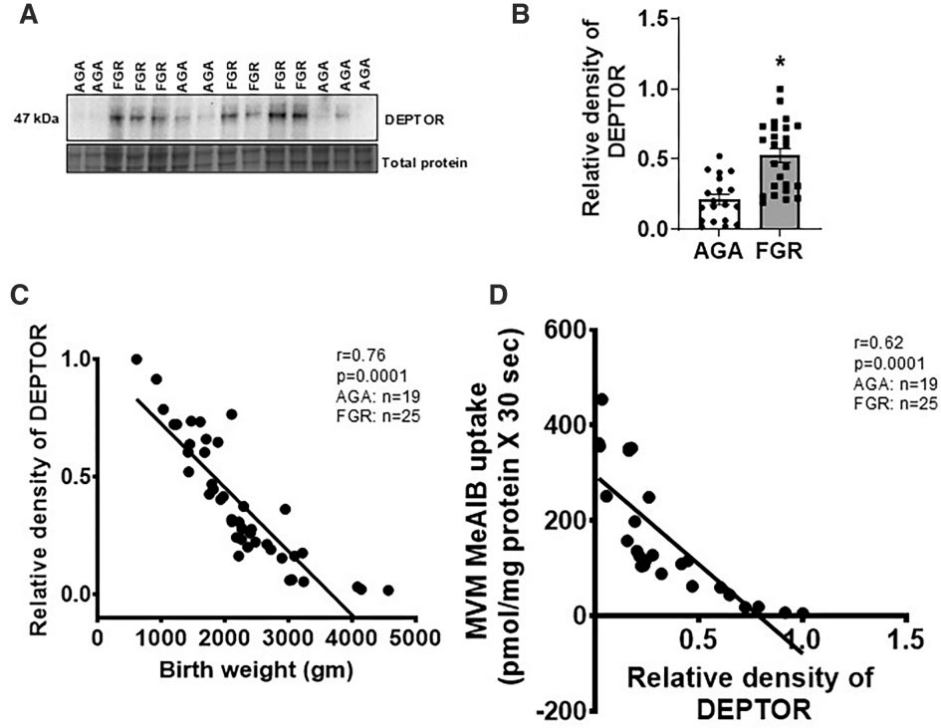
Placental DEPTOR protein expression in human fetal growth restriction (FGR). (A) Representative western blot of DEPTOR expression in placental homogenates of appropriate-for-gestational age (AGA) and FGR groups. (B) Relative expression of DEPTOR in placental homogenates of AGA and FGR. After normalization to total protein, the mean density of AGA samples was assigned an arbitrary value of 1. Subsequently, individual FGR density values were expressed relative to this mean. AGA, *n* = 19; FGR, *n* = 25.

First, we investigated the relationship between placental DEPTOR protein expression and birth weight and MVM system A amino acid transport activity in placentas collected from normal healthy appropriately grown for gestational age (AGA) and FGR pregnancies (Table 1). We previously reported that mTOR signaling and placental system A amino acid transport activity in the same human IUGR cohort placenta used in the current study were reported to be downregulated.^8^ The protein expression of DEPTOR was significantly higher (*P* = .0001; AGA, *n* = 19; FGR, *n* = 25) in FGR placentas as compared to AGA (Figure 2A and B). Placental DEPTOR protein expression in placental homogenates of AGA and FGR pregnancies were both inversely correlated with birth weight (*r* = 0.76, *P* = .0001, AGA, *n* = 19; FGR, *n* = 25;Figure 2C) and MVM system A amino acid transport activity (*r* = 0.62, *P* = .0001, AGA, *n* = 19; FGR, *n* = 25; Figure 2D).

**Table 1.** Selected Clinical Data^a^

	Control (*n* = 19)	FGR (*n* = 25)
Maternal age (years)	25.9 ± 1.29	28.7 ± 1.23
BMI (kg/m^2^)	28.3 ± 2.6	26.8 ± 2.0
Gestational age (weeks)	33.9 ± 0.95	35.7 ± 0.61
Birth weight (g)	2493 ± 236	1804 ± 110^b^
Birth weight percentile^c^	55.9 ± 4.6	2.4 ± 0.3^d^
Placental weight (g)	566 ± 42.0	394 ± 18.4^e^
Fetal sex (M/F)	7/12	8/17
Mode of delivery (C/V)	6/13	15/10

Abbreviations: C, caesarean section; F, female; M, male; *n*, numbers; V, vaginal delivery.

a Data are presented as means ± SEM.

b *P* < .05.

c By corresponding gestational age and fetal sex.

d *P* < .0001.

e *P* < .01.

### Trophoblast-Specific Knockdown of *Deptor* in Mice

**Figure 3.**
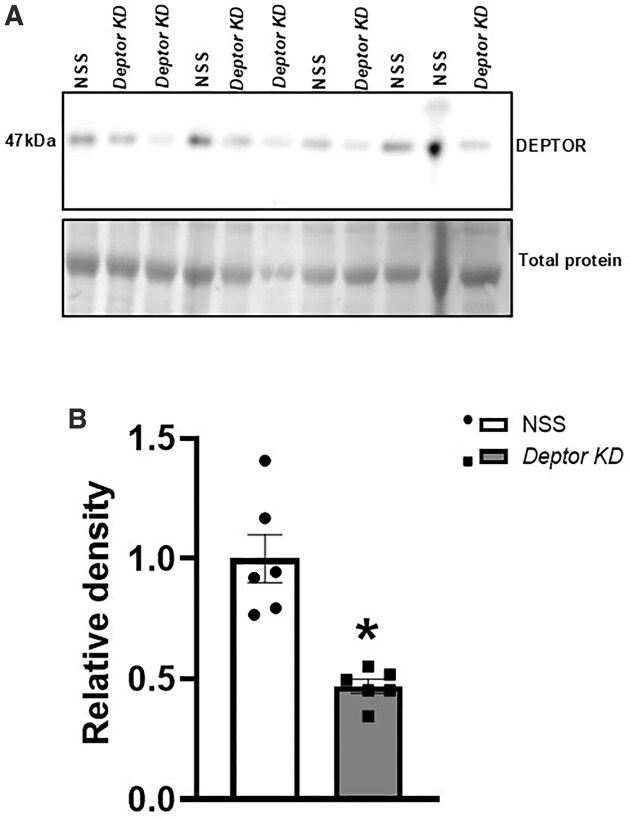
Placental DEPTOR protein expression following trophoblast-specific *Deptor* knockdown in mice. (A and B) DEPTOR protein expression in NSS and *Deptor* KD placentas, pooled from *n* = 6 litters in each group (NSS, 23 placentas; *Deptor* KD, 23 placentas). At E18.5, animals were sacrificed, and placentas from each genotype were collected, pooled, and homogenized. DEPTOR protein expression was determined by immunoblotting. (A) Representative western blot of DEPTOR expression in placental homogenates of NSS and *Deptor* KD placentas. Equal loading was performed. (B) Summary of the western blot data. *n* = 6 litters in each group. Each dot represents litter mean value for respective genotype. Values are expressed as means ± SEM. All placentas of each genotype in a litter were pooled prior to analysis. *P < .05 vs. NSS; paired Student’s *t*-test. NSS, nonsense sequence (control) group.

At E18.5, implantation and viability rates were comparable in the NSS and *Deptor* knockdown (*Deptor* KD) placentas (data not shown). Placental *Deptor* mRNA expression was 47% lower (*P* = .0001, *n* = 6 litters in each group) in *Deptor* knockdown compared to NSS placentas (Figure S1a). Each placenta was dissected to separate the nutrient-transporting labyrinthine zone from the hormone-producing junctional zone to measure the *Deptor* mRNA expression. As shown in Figure S1b and c, *Deptor* mRNA expression in both labyrinth (−49%, *P* = .03) and junctional zones (−39%, *P* = .0003) was reduced in *Deptor* KD (*n* = 5–6 litters in each group). Likewise, *Deptor* knockdown reduced the placental DEPTOR protein expression by 53% (*P* = .003, *n* = 6) compared to NSS (Figure 3A and B). There was no significant difference in *Deptor* mRNA expression in the fetal liver (*P* = .34), heart (*P* = .78), brain (*P* = .50), lung (*P* = .85), kidney (*P* = .92), or muscle (*P* = .77) between the NSS and *Deptor* KD (*n* = 6 litters in each group) groups, as shown in Figure S2a, b, c, d, e and f. In addition, *Deptor* mRNA expression in the fetal membrane (*P* = .35) and decidua (*P* = .78) was comparable between NSS and *Deptor* KD groups (*n* = 6; Figure S3a and b). This finding further supports that the targeted gene manipulation was specific to trophoblast cells.

### Trophoblast-Specific Knockdown of *Deptor* Increases Placental and Fetal Weight in Mice

**Figure 4.**
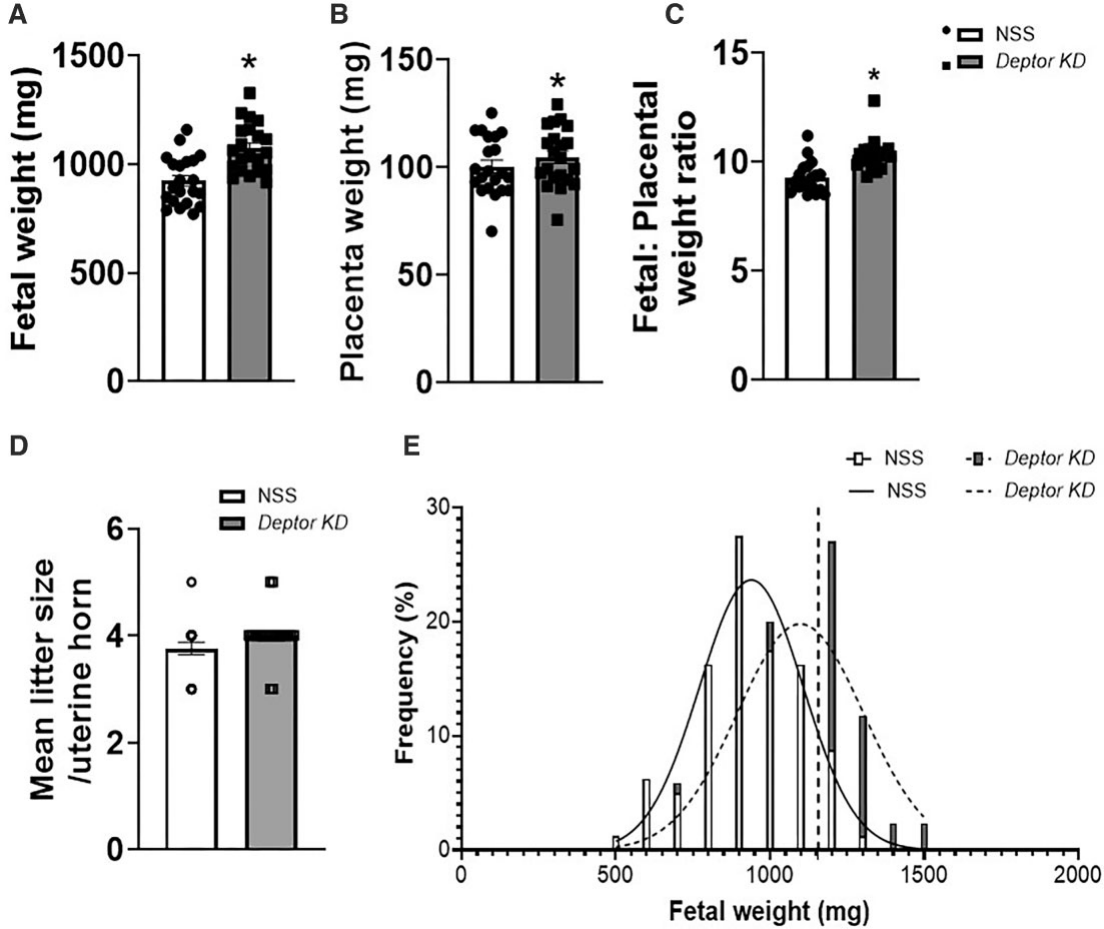
Fetal and placental weight, fetal:placental (F:P) weight ratio, litter size, and fetal weight distribution curves following trophoblast-specific *Deptor* knockdown in mice. (A–D) Compared to NSS mice, trophoblast-specific *Deptor* knockdown mice exhibit increased (A) fetal weights and (B) placental weights at embryonic day 18.5. Trophoblast-specific *Deptor* knockdown in mice increased (C) fetal:placental weight ratio but not (D) litter size. (A–C) Each dot represents litter mean value. Values are expressed as means ± SEM. (A and B) *P < .05 vs. NSS, paired Student’s *t*-test. (C and D) *P < .05 vs. NSS; paired Wilcoxon matched-pairs signed rank test. (E) Frequency distribution of individual fetal weights in NSS (*n* = 83), and *Deptor* KD (*n* = 88) dams. Mean fetal weight of trophoblast-specific *Deptor* KD mice (dashed line, *r*^2^ = 0.71; *n* = 88 fetuses, 22 litters) was significantly higher than in NSS (NS, solid line, *r*^2^ = 0.91; *n* = 83 fetuses, 22 litters) mice. The vertical dashed line represents the 90th centile on the NSS curve (1156 mg), and 43% of *Deptor* KD fetuses fall above this. Gaussian curves fitted by least-squares nonlinear regression and compared by the extra sum-of-squares *F*-test (*P* = .01). NSS, nonsense sequence (control) group.

Figure 4A demonstrates that trophoblast-specific knockdown of *Deptor* resulted in a 16% increase in fetal weight (*P* = .0001, *n* = 22 litters; Figure S4a, *n* = 83–88 fetuses/group) as compared to the NSS group. In addition, placental weight was increased by 4.5% (Figure 4B, *P* = .01, *n* = 22 litters; Figure S4b, *n* = 83–88 placentas/group) following trophoblast-specific *Deptor* KD. As shown in Figure 4C and Figure S4c, the fetal:placental weight ratio was significantly increased by 11.5% (*P* = .0001, *n* = 22 litters and *n* = 83–88 fetuses and placentas/group) as a result of trophoblast-specific *Deptor* knockdown as compared to the NSS group. There was no statistically significant difference in the number of pups per uterine horn between *Deptor* KD (4.0 ± 0.09 pups/uterine horn) and NSS (3.8 ± 0.11 pups/uterine horn) groups (Figure 4D, *P* = .125, *n* = 22 litters). To characterize the observed fetal overgrowth in more detail, fetal weight distribution curves were plotted for NSS and *Deptor* KD groups (Figure 4E). The fetal weight distribution following trophoblast-specific *Deptor* KD was shifted to the right (indicative of fetal overgrowth) and 43% of the fetuses had a weight above the 90th centile (1156 mg) following trophoblast-specific *Deptor* KD as compared to the fetal weight distribution in the NSS group.

### Trophoblast-Specific Knockdown of *Deptor* Activates Placental Mtorc1 and Mtorc2 Signaling

**Figure 5.**
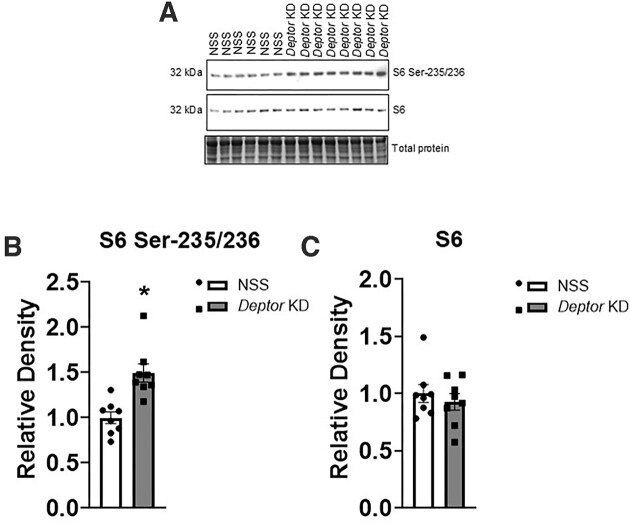
Placental mTORC1 signaling activity following trophoblast-specific *Deptor* knockdown in mice. At E18.5, animals were sacrificed, and placentas from each litter were weighed, pooled, and homogenized. (A–C) Activation of placental mTORC1 signaling following trophoblast-specific *Deptor* knockdown. The phosphorylation and total protein expression of key intermediate in the mTORC1 signaling pathway was determined by immunoblotting. (A) Representative western blot of S6 Serine-235/236 and S6 in placental homogenates of NSS and *Deptor* KD placentas. Equal loading was performed. (B and C) Summary of the western blot data. Each dot represents litter mean value (*n* = 8 litters representing 29 NSS conceptuses and 32 *Deptor* KD conceptuses). Values are expressed as means ± SEM. All placentas of each genotype in a litter were pooled prior to analysis. Litter mean values for NSS and *Deptor* KD conceptuses compared by paired Student’s *t*-test. NSS, nonsense sequence (control).

**Figure 6.**
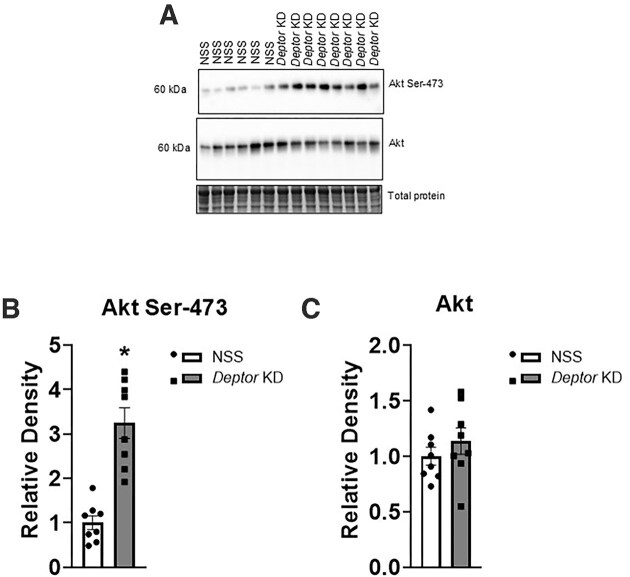
Placental mTORC2 signaling activity following trophoblast-specific *Deptor* knockdown in mice. At E18.5, animals were sacrificed, and placentas from each litter were weighed, pooled, and homogenized. (A–C) Activation of placental mTORC2 signaling following trophoblast-specific *Deptor* knockdown. The phosphorylation and total protein expression of key intermediate in the mTORC2 signaling pathway was determined using immunoblotting. (A) Representative western blot of Akt Serine-473 and Akt in placental homogenates of NSS and *Deptor* KD placentas. Equal loading was performed. (B and C) Summary of the western blot data. Each dot represents litter mean value (*n* = 8 litters represent 29 NSS conceptuses and 32 *Deptor* KD conceptuses). Values are expressed as means ± SEM. All placentas of each genotype in a litter were pooled prior to analysis. Litter mean values for NSS and *Deptor* KD conceptuses compared by paired Student’s *t*-test. NSS, nonsense sequence (control) group.

Next, we determined the functional readouts of the mTOR signaling pathways in NSS and *Deptor* KD placentas. The knockdown of *Deptor* significantly increased (+50%, *n* = 8 litters, *P* = .0006; Figure 5A and B) S6 ribosomal protein phosphorylation at Serine-235/236, an mTORC1 downstream target. Akt is a key downstream target of mTORC2^60^ and we used the phosphorylation of Akt at Serine-473 as a readout for mTORC2 activity. Trophoblast-specific *Deptor* knockdown increased the phosphorylation of protein kinase B (Akt-Ser 473, +220%, *P* = .001, *n* = 8 litters; Figure 6A and B), compared to NSS. However, total S6 (S6, *P* = .339, *n* = 8 litters; Figure 5A–C) and Akt (Akt, *P* = .379, *n* = 8 litters; Figure 6A–C) expressions were comparable between NSS and *Deptor* KD groups.

### Trophoblast-Specific Knockdown of *Deptor* Increases Placental TPM LAT1 and SNAT2 Expression

**Figure 7.**
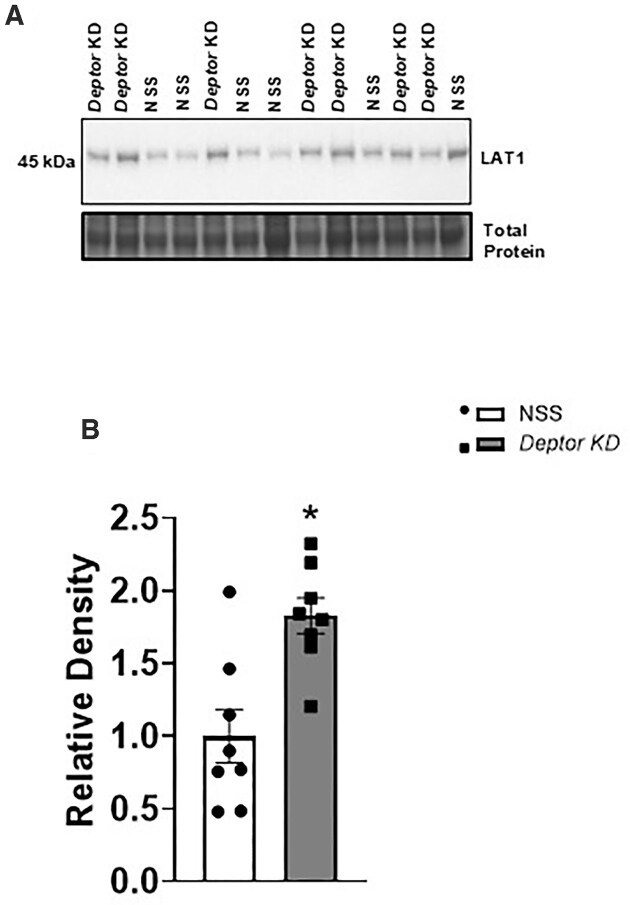
Placental trophoblast plasma membrane (TPM) LAT1 expression following trophoblast-specific *Deptor* knockdown in mice. At E18.5, animals were sacrificed, and placentas from each litter were weighed, pooled, and homogenized. (A and B) Increased TPM LAT1 expression following Deptor knockdown in mice. TPMs were isolated, and the protein expression of the amino acid transporter isoform LAT1 (system L) was determined using Western blot. (A) Representative western blots of LAT1 expression in TPM of NSS and *Deptor* KD placentas. Equal loading was performed. (B) Summary of the western blot data. Each dot represents litter mean value (*n* = 8 litters represent 29 NSS conceptuses and 32 *Deptor* KD conceptuses). Values are expressed as means ± SEM. All placentas of each genotype in a litter were pooled prior to the isolation of TPM. Litter mean values for NSS and *Deptor* KD conceptuses compared by paired Student’s *t*-test. NSS, nonsense sequence (control) group.

**Figure 8.**
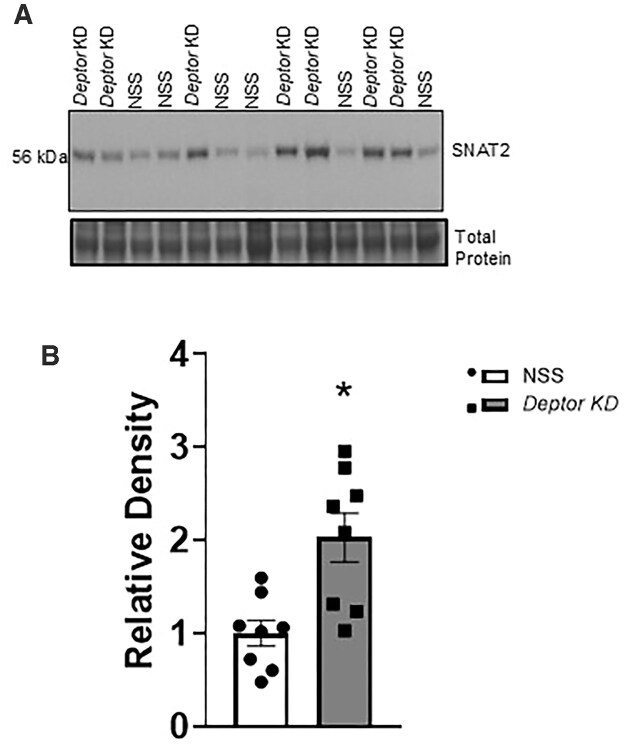
Placental trophoblast plasma membrane (TPM) SNAT2 expression following trophoblast-specific *Deptor* knockdown in mice. At E18.5, animals were sacrificed, and placentas from each litter were weighed, pooled, and homogenized. (A and B) Increased TPM SNAT2 expression following *Deptor* knockdown in mice. TPMs were isolated, and the protein expression of the amino acid transporter isoform SNAT2 (system A) was determined using western blot. (A) Representative western blots of SNAT2 expression in TPM of NSS and *Deptor* KD placentas. Equal loading was performed. (B) Summary of the western blot data. Each dot represents litter mean value (*n* = 8 litters represent 29 NSS conceptuses and 32 *Deptor* KD conceptuses). Values are expressed as means ± SEM. All placentas of each genotype in a litter were pooled prior to isolation of TPM. Litter mean values for NSS and *Deptor* KD conceptuses compared by paired Student’s *t*-test. NSS, nonsense sequence (control) group.

The LAT1 and SNAT2 transporter must be translocated to the TPM in order to facilitate the cellular uptake and transplacental transport of amino acids. We previously demonstrated that mTOR signaling regulates the LAT1 and SNAT2 transporter membrane trafficking in PHT cells at the post-translational level.^8,46,47,55^ To determine the changes in plasma membrane LAT1 and SNAT2 expression, we isolated TPM from NSS and *Deptor* KD placentas and performed western blotting. As shown in Figures 7 and 8, TPM LAT1 (+82%, *P* = .0001, *n* = 8 litters) and SNAT2 (+99%, *P* = .0001, *n* = 8 litters) protein expression was significantly higher in *Deptor* KD placentas than in NSS placentas.

### Trophoblast-Specific Knockdown of *Deptor* Increases Placental TPM System L and System A Activity

**Figure 9:**
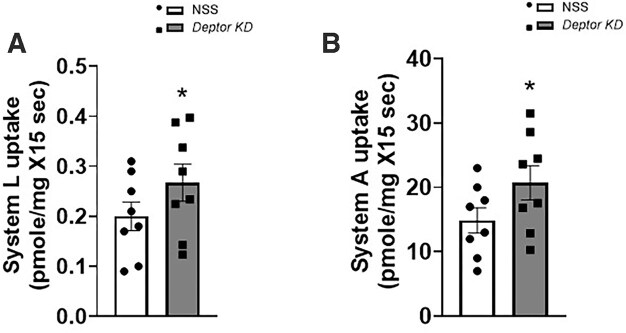
Placental trophoblast plasma membrane (TPM) system L and A activities following trophoblast-specific *Deptor* knockdown in mice. At E18.5, animals were sacrificed, and placentas from each litter were weighed, pooled, and homogenized. TPMs were isolated. System L (A) and system A (B) transporter activities were determined using isotope-labeled substrates and rapid filtration techniques in TPM isolated from NSS and *Deptor* KD placenta at E18.5. Each dot represents litter mean value (*n* = 8 litters represent 29 NSS conceptuses and 32 *Deptor* KD conceptuses). Values are expressed as means ± SEM. All placentas of each genotype in a litter were pooled prior to isolation of TPM. Litter mean values for NSS and *Deptor* KD conceptuses compared by paired Student’s *t*-test. NSS, nonsense sequence (control) group.

In TPM isolated from *Deptor* KD placentas, the capacity to transport leucine was increased (+30%, *P* = .0002, *n* = 8 litters; Figure 9A) compared to TPM isolated from NSS placentas. Moreover, as shown in Figure 9B, knockdown of *Deptor* in the trophoblast increased TPM system A amino acid transport by 39% (*P* = .003, *n* = 8 litters) compared to TPM isolated from NSS placentas.

### Trophoblast-Specific Knockdown of *Deptor* Increases Transplacental System L and A Amino Acid Transport Activity

**Figure 10.**
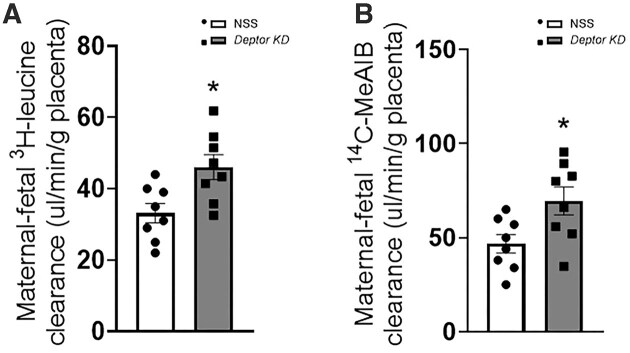
In vivo transplacental amino acid transport following trophoblast-specific *Deptor* knockdown in mice. Unidirectional maternal-fetal clearances for (A) ^3^H-leucine and (B) ^14^C-MeAIB were measured in anesthetized dams. Each dot represents litter mean value (*n* = 8 litters represent 31 NSS conceptuses and 33 *Deptor* KD conceptuses). Values are expressed as means ± SEM. Litter mean values for NSS and Deptor KD conceptuses compared by paired Student’s *t*-test. NSS, nonsense sequence (control) group.

To examine whether the increased fetal growth in response to trophoblast-specific *Deptor* knockdown was associated with increased in vivo transplacental amino acid transport, unidirectional maternofetal clearance of ^3^H-leucine (system L–mediated transport) and ^14^C-MeAIB (system A amino acid transport) was measured. Trophoblast-specific *Deptor* knockdown increased the transplacental transport of ^3^H-leucine (+39%, *P* = .0001, *n* = 8 litters; Figure 10A) and ^14^C-MeAIB (+49%, *P* = .002, *n* = 8 litters; Figure 10B) compared to the NSS group.

### Trophoblast-Specific Knockdown of *Deptor* Did Not Induce Cellular Senescence in the Placenta

There is also evidence suggesting that mTORC1 signaling plays a role in cellular senescence in other cell types^61^. Here, we examined whether trophoblast-specific *Deptor* knockdown was associated with cellular senescence in the placenta. We measured the key cellular senescence marker p53 (transcription factor plays a critical role in cellular responses to stress) expression in the NSS and *Deptor* KD placentas. As shown in Figure S5, p53 expression was comparable between NSS and *Deptor* KD placentas, suggesting that trophoblast-specific knockdown of *Deptor* did not induce cellular senescence in the placenta.

## Discussion

This study demonstrates for the first time that the inhibition of placental *Deptor* signaling in vivo is mechanistically linked to increased placental mTORC1 and mTORC2 signaling and transplacental amino acid transport capacity, and results in fetal overgrowth. Furthermore, we provide evidence that the findings in mice have clear clinical relevance by demonstrating that human placental DEPTOR protein expression is significantly decreased in FGR, and placental DEPTOR protein expression is negatively correlated with birth weights and MVM system A activity.

The effect of trophoblast-specific *Deptor* knockdown in this study mirrors findings in a mouse model of maternal obesity.^39,62^ In this model, activation of placental mTORC1 and mTORC2 signaling and increased transplacental system L and system A amino acid transport were associated with fetal overgrowth.^39^,^62^ Likewise, administering IGF-1 (Insulin-like growth factor 1) directly into the amniotic fluid of pregnant sheep increases fetal weight and the transport of system L amino acids.^38^ Furthermore, in a rat model of GDM (Gestational Diabetes Mellitus) programmed in utero placental mTOR signaling was reported to be activated and fetal growth increased.^63^ Shang and Wen^43^ reported that the activity of placental mTOR signaling positively correlated with birth weight in women with GDM. Similarly, placental mTORC1 and mTORC2 signaling activity was reported to be increased in women with GDM and LGA (Large for gestational age) infants than normal pregnant women.^19^ We also previously demonstrated that activation of placental mTOR signaling is associated with increased placental system A amino acid transport and fetal overgrowth in obese women.^10^ Maternal probiotic supplementation during late pregnancy in the sow activated placental mTOR signaling and upregulated the expression of amino acid transporters LAT1 and SNAT1 in the placenta^64^. Conversely, pregnant mice with FGR induced by rapamycin show smaller placentas and decreased mTOR activity.^32,65^ Placentas from human FGR pregnancies had lower phosphorylated Akt and lower mTOR phosphorylation compared to that of normal pregnant women.^19^ Akhaphong et al.^34^ demonstrated that manipulating mTOR signaling in the placenta, either by decreasing or increasing its activity, influences the long-term metabolic health of the offspring. Moreover, Fahlbusch^66^ and coworkers reported that the level of activated placental mTOR correlates with early catch-up growth following FGR in human pregnancy. We propose that fetal overgrowth observed in our study was due to the activation of placental mTORC1 and mTORC2 signaling by reduction in *Deptor* mRNA expression, which resulted in an activation of transplacental system L and A amino acid transport. This change in placental transport capacity would increase fetal amino acid availability and stimulate fetal growth.

Placental mTORC1 and mTORC2 signaling pathways are positive regulators of system L and A amino acid transporters in cultured PHT cells and human placental villous explants.^15,55,67^ Using gene silencing approaches, we previously demonstrated that silencing of DEPTOR protein expression in PHT cells activates both mTORC1 and mTORC2 signaling.^46,47^ Furthermore, activation of both mTORC1 and mTORC2 signaling increases trophoblast system A and L amino acid transporter activity by increasing the plasma membrane trafficking of specific system A (SNAT2, *SLC38A2*) and system L (LAT1, *SLC7A5*) isoforms with no effect on overall protein expression.^46,47^ mTORC1 and 2 signaling regulates the SNAT2 and LAT1 trafficking via at least 2 distinct mechanisms.^46,47^ Inhibition of mTORC1 signaling causes upregulation of the Nedd4-2 E3 ubiquitin ligase, which induces ubiquitination of SNAT2 and LAT1 that promotes the withdrawal of transporters from the plasma membrane and transport to lysosome for degradation.^47^ Thus, it is possible that inhibition of placental mTORC1 activity in human FGR directly contributes to the reduced fetal growth in this pregnancy complication by limiting fetal amino acid availability mediated by decreased placental system A and L activity.^8^ Inhibition of mTORC2 signaling in human primary trophoblast cells markedly decreased the trafficking of LAT1 and SNAT2 to the plasma membrane and decreased system L and A transport by downregulating Rho-GTPases Cdc42 and Rac.^46^ Placental Cdc42 and Rac1 protein expression was downregulated in human FGR and was positively correlated with placental mTORC2 signaling.^46^ In this study, we found that trophoblast-specific *Deptor* knockdown increased the abundance of system L amino acid transporter isoform LAT1 and system A amino acid transporter isoform SNAT2 in the TPM, providing mechanistic in vivo evidence of DEPTOR signaling controlling TPM trafficking of amino acid transporters.

In this study, we observed a small but significant increase (4.5%) in placental weight as a result of placenta-specific *Deptor* knockdown. It is possible that increased placental amino acid uptake following decreased *Deptor* expression and activation of mTOR signaling contributes to this increased placental growth. In support of this proposal, arginine availability is linked to increasing placental weight, as it plays a key role in placental growth and development by facilitating protein synthesis and supporting vascularization within the placenta through the production of nitric oxide.^68,69^ Moreover, maternal leucine supplementation increased placental weight in pregnant rats^70^ and promoted the development of offspring in sows by increasing the placental expression of amino acid transporters LAT1 and SNAT1 and protein synthesis through the PI3K/Akt/mTOR signaling pathway^71^. Thus, one explanation for the increased placental weight in the *Deptor* knockdown group may be increased leucine and arginine availability that stimulates protein synthesis and ultimately contribute to increased placental weight.

By assessing transplacental amino acid transport with radiolabeled amino acids, we confirmed that trophoblast-specific in vivo *Deptor* knockdown causes a marked increase in system L and A amino acid transporter activity. Specifically, *Deptor* knockdown increased system L–mediated transplacental transport by 39% and placental system A activity by 49%. Amino acid transport regulates trophoblast functionality. For example, taurine transport in trophoblast cells has a role in trophoblast fusion/differentiation and multinucleation^72^. Arginine transport promoted cell cycle, and the mRNA expressions of IGF-1, and increased cellular protein synthesis rate, as well as estradiol and hCG (human chorionic gonadotropin) secretion, and immune function in porcine trophoblast cells.^73^ Furthermore, arginine reduced trophoblast apoptosis, improved placental function, and promoted fetal development.^74^ We propose that increased transplacental transport of amino acids supports placental function and accelerated fetal growth following trophoblast-specific *Deptor* knockdown in our model. This aligns with our recent study demonstrating that placental-specific *Slc7a5* overexpression in mice results in increased transporter protein expression of LAT1 in the TPM, which increased the transplacental system L–mediated supply of essential amino acids to the fetal circulation.^48^ This is similar to the finding that human placenta microvillus membrane system A activity is increased in placentas from large-for-gestational-age babies of obese women.^10^

In this study, we utilized lentivirus-mediated transduction to generate a mouse with trophoblast-specific knockdown of *Deptor*. Trophoblast-specific targeting was validated by unaltered *Deptor* expression in uterus, decidua, fetal membranes, and organs (liver, heart, brain, lung, and skeletal muscle). As a result of trophoblast-specific knockdown of *Deptor*, the expression of *Deptor* mRNA was decreased in both the labyrinth and the junctional zone of the placenta. Notably, this reduction in trophoblast *Deptor* mRNA level resulted in activation of mTORC1 and C2 signaling and nutrient transport, and fetal overgrowth. Thus, activation of mTOR signaling by knockdown the endogenous mTOR inhibitor *Deptor* specifically in the trophoblast had the same effect on nutrient transport and fetal growth as increasing mTORC1 and C2 signaling levels by trophoblast-specific overexpression of the *Slc7a5*.^48^ Gouvêa et al^75^ demonstrated that methionine supplementation during mid-gestation in bovine pregnancy resulted in upregulation of placental mTOR as well as the expression of amino acid transporters *SLC1A5, SLC7A5, SLC38A6*, and *SLC38A11* and fetal overgrowth in male offspring. Similarly, methionine supplementation during late gestation in dairy cows upregulated the placental mTOR, as well as the amino acid transporters *SLC1A5, SLC7A5, SLC38A6*, and *SLC38A11* mRNA expression and increased fetal growth.^76^ In contrast, we have recently shown that using piggyBac transposase-mediated inducible trophoblast-specific knockdown of *Mtor* in late pregnancy decreases placental nutrient transport and results in reduced fetal weight in mice.^77^ Rapamycin-mediated mTOR inhibition decreased the *SLC38A*4 mRNA expression in the primary ovine trophoblast cells.^78^ In addition, placental mTORC1 activity and the mature form of the amino acid transporter SNAT2 were also reduced in the women delivering small-for-gestational-age babies.^79^ Furthermore, overexpression of *Adipor2* specifically in the trophoblast downregulated placental mTORC1 signaling and nutrient transport, resulting in FGR.^50^ These findings therefore provide solid mechanistic evidence that trophoblast DEPTOR-mTOR signaling directly influences placental function and fetal growth.

Our findings in the placenta-specific *Deptor* KD mouse model suggest that lower placental expression of DEPTOR, an endogenous inhibitor of mTOR signaling, is associated with increased mTOR signaling, nutrient transport, and placental and fetal weight. To demonstrate the clinical relevance of our findings, we determined placental DEPTOR protein expression in human FGR. We have previously reported that FGR in this cohort is associated inhibition of placental mTOR signaling and decreased system A amino acid activity.^8^ Here, we reported that placental DEPTOR protein expression was elevated in the human FGR placentas and inversely correlated with birth weight and placental system A amino acid transport activity. Collectively, these observations are consistent with the possibility that increased placental DEPTOR protein expression in FGR is mechanistically linked to placental mTOR inhibition, decreased placental amino acid transport, and reduced fetal growth. Further supporting the translational significance of our findings in the current study, placental mTOR signaling is increased in pregnancy complications associated with fetal overgrowth.^10^ Maternal obesity and GDM, which increase the risk of fetal overgrowth, have been reported to be associated with increased placental amino acid transport capacity.^10,43,63^ Specifically, system L activity in syncytiotrophoblast MVM was positively correlated with birth weight in a cohort of normal and obese women.^10^ Transplacental amino acid transport mediated by system L and A activity is increased in a mouse model of maternal obesity linked with fetal overgrowth.^39^ Thus, the mechanistic link between activation of placental mTOR signaling, increased placental amino acid transport activity, and fetal overgrowth reported here in the *Deptor* knockdown mouse model provides novel insights into the causes of human pregnancy complications associated with fetal overgrowth. One of the limitations of this study is that we did not study the sex-specific effect of placental *Deptor* signaling on fetal growth and nutrient transport.

The placental mTOR signaling pathway is a key nutrient sensor. It responds to an array of maternal metabolic and nutritional signals and is a crucial regulator of placental function, encompassing nutrient transport, mitochondrial activity, and protein synthesis. We suggest that placental mTOR signaling serves as an essential hub linking uteroplacental blood flow, maternal nutrition, and metabolism to changes in placental function, fetal growth, and developmental programming. For instance, when the maternal supply line cannot adequately support normal fetal growth due to undernutrition or reduced uteroplacental blood flow, placental DEPTOR signaling is activated, which leads to mTOR inhibition. This mTOR inhibition results in the downregulation of several vital placental functions, directly contributing to decreased fetal growth. We speculate that this control system evolved under the pressures of starvation, aimed at protecting the mother at the expense of a smaller fetus. We also contend that this regulatory mechanism responds to nutritional inputs in the opposite direction; for example, in cases of maternal obesity or gestational diabetes, placental inhibition of DEPTOR signaling may activate placental mTOR signaling and contribute to fetal overgrowth in some of these pregnancies. Thus, our current report, which establishes a direct mechanistic link between placental DEPTOR signaling, nutrient transport, and fetal growth in mice, supports this model.

In conclusion, our study provides mechanistic evidence that in vivo trophoblast-specific knockdown of *Deptor* causes fetal overgrowth by increasing placental mTORC1 and mTORC2 signaling and amino acid transport. We propose that activation of placental mTOR signaling and increased nutrient transport in maternal obesity directly contributes to fetal overgrowth^39,62^ and programs the offspring for cardiometabolic disease mediated by a direct effect on placental function.,^80, 81^ We speculate that the placental *Deptor* may be a therapeutic target to normalize placental function and fetal growth and alleviate fetal programming of disease in pregnancies complicated by FGR or fetal overgrowth.

## Supplementary Material

zqaf018_Supplemental_Files

## Data Availability

All supporting data and associated protocols for this manuscript are available upon request.

## References

[1] Gluckman PD, Hanson MA, Cooper C, Thornburg KL. Effect of in utero and early-life conditions on adult health and disease. N Engl J Med. 2008;359(1):61–73. 18596274 10.1056/NEJMra0708473 PMC3923653

[2] Gluckman PD, Hanson MA. Living with the past: evolution, development, and patterns of disease. Science. 2004;305(5691):1733–1736. 15375258 10.1126/science.1095292

[3] Gluckman PD, Hanson MA. The developmental origins of the metabolic syndrome. Trends Endocrinol Metab. 2004;15(4):183–187. 15109618 10.1016/j.tem.2004.03.002

[4] Barker DJP, Godfrey KM, Gluckman PD, Harding JE, Owens JA, Robinson JS. Fetal nutrition and cardiovascular disease in adult life. Lancet. 1993;341(8850):938–941. 8096277 10.1016/0140-6736(93)91224-a

[5] Maiiendran D, Donnai P, Glazier JD, D'souza SW, Boyd RDH, Sibley CP. Amino acid (system A) transporter activity in microvillous membrane vesicles from the placentas of appropriate and small for gestational age babies. Pediatr Res. 1993;34(5):661–665. 8284106 10.1203/00006450-199311000-00019

[6] Glazier JD, Cetin I, Perugino G et al. Association between the activity of the system A amino acid transporter in the microvillous plasma membrane of the human placenta and severity of fetal compromise in intrauterine growth restriction. Pediatr Res. 1997;42(4):514–519. 9380446 10.1203/00006450-199710000-00016

[7] Jansson T, Scholtbach V, Powell TL. Placental transport of leucine and lysine is reduced in intrauterine growth restriction. Pediatr Res. 1998;44(4):532–537. 9773842 10.1203/00006450-199810000-00011

[8] Chen Yi-Y, Rosario FJ, Shehab MA, Powell TL, Gupta MB, Jansson T. Increased ubiquitination and reduced plasma membrane trafficking of placental amino acid transporter SNAT-2 in human IUGR. Clin Sci (Colch). 2015;129(12):1131–1141. 26374858 10.1042/CS20150511 PMC4614027

[9] Jansson T, Ekstrand Y, BjöRn C, Wennergren M, Powell TL. Alterations in the activity of placental amino acid transporters in pregnancies complicated by diabetes. Diabetes. 2002;51(7):2214–2219. 12086952 10.2337/diabetes.51.7.2214

[10] Jansson N, Rosario FJ, Gaccioli F et al. Activation of placental mTOR signaling and amino acid transporters in obese women giving birth to large babies. J Clin Endocrinol Metab. 2013;98(1):105–113. 23150676 10.1210/jc.2012-2667 PMC3537112

[11] Castillo-Castrejon M, Yamaguchi K, Rodel RL et al. Effect of type 2 diabetes mellitus on placental expression and activity of nutrient transporters and their association with birth weight and neonatal adiposity. Mol Cell Endocrinol. 2021;532:111319. 33989714 10.1016/j.mce.2021.111319 PMC8206039

[12] Laplante M, Sabatini DM. mTOR signaling in growth control and disease. Cell. 2012;149(2):274–293. 22500797 10.1016/j.cell.2012.03.017 PMC3331679

[13] Sarbassov DD, Ali SM, Sabatini DM. Growing roles for the mTOR pathway. Curr Opin Cell Biol. 2005;17(6):596–603. 16226444 10.1016/j.ceb.2005.09.009

[14] Sabatini DM . mTOR and cancer: insights into a complex relationship. Nat Rev Cancer. 2006;6(9):729–734. 16915295 10.1038/nrc1974

[15] Roos S, Jansson N, Palmberg I, Säljö K, Powell TL, Jansson T. Mammalian target of rapamycin in the human placenta regulates leucine transport and is down-regulated in restricted fetal growth. J Physiol. 2007;582(pt 1):449–459. 17463046 10.1113/jphysiol.2007.129676 PMC2075295

[16] Yung H-Wa, Calabrese S, Hynx D et al. Evidence of placental translation inhibition and endoplasmic reticulum stress in the etiology of human intrauterine growth restriction. Am J Pathol. 2008;173(2):451–462. 18583310 10.2353/ajpath.2008.071193 PMC2475782

[17] Hung T-Ho, Hsieh T'-T', Wu C-Pu, Li M-J, Yeh Yi-L, Chen S-Fu. Mammalian target of rapamycin signaling is a mechanistic link between increased endoplasmic reticulum stress and autophagy in the placentas of pregnancies complicated by growth restriction. Placenta. 2017;60:9–20. 29208245 10.1016/j.placenta.2017.10.001

[18] Dimasuay KG, Aitken EH, Rosario F et al. Inhibition of placental mTOR signaling provides a link between placental malaria and reduced birthweight. BMC Med. 2017;15(1):1. 28049467 10.1186/s12916-016-0759-3 PMC5209943

[19] Hung TH, Wu CP, Chen SF. Differential changes in Akt and AMPK phosphorylation regulating mTOR activity in the placentas of pregnancies complicated by fetal growth restriction and gestational diabetes mellitus with large-for-gestational age infants. Front Med. 2021;8:788969. 10.3389/fmed.2021.788969 PMC8685227 34938752

[20] Zheng W, Zhang Y, Xu P et al. TFEB safeguards trophoblast syncytialization in humans and mice. Proc Natl Acad Sci USA. 2024;121(28):e2404062121. 38968109 10.1073/pnas.2404062121 PMC11253012

[21] Tsai K, Tullis B, Jensen T, Graff T, Reynolds P, Arroyo J. Differential expression of mTOR related molecules in the placenta from gestational diabetes mellitus (GDM), intrauterine growth restriction (IUGR) and preeclampsia patients. Reprod Biol. 2021;21(2):100503. 33826986 10.1016/j.repbio.2021.100503

[22] Xu J, Wang J, Cao Y et al. Downregulation of placental amino acid transporter expression and mTORC1 signaling activity contributes to fetal growth retardation in diabetic rats. Int J Mol Sci. 2020;21(5):1849. 32156054 10.3390/ijms21051849 PMC7084659

[23] Rosario FJ, Jansson N, Kanai Y, Prasad PD, Powell TL, Jansson T. Maternal protein restriction in the rat inhibits placental insulin, mTOR, and STAT3 signaling and down-regulates placental amino acid transporters. Endocrinology. 2011;152(3):1119–1129. 21285325 10.1210/en.2010-1153 PMC3858644

[24] Kavitha JV, Rosario FJ, Nijland MJ et al. Down-regulation of placental mTOR, insulin/IGF-I signaling, and nutrient transporters in response to maternal nutrient restriction in the baboon. FASEB J. 2014;28(3):1294–1305. 24334703 10.1096/fj.13-242271 PMC3929672

[25] Arroyo JA, Brown LD, Galan HL. Placental mammalian target of rapamycin and related signaling pathways in an ovine model of intrauterine growth restriction. Am J Obstet Gynecol. 2009;201(6):616.e1–616.e7. 10.1016/j.ajog.2009.07.031 PMC2789863 19800600

[26] Rosario FJ, Schumacher MA, Jiang J, Kanai Y, Powell TL, Jansson T. Chronic maternal infusion of full-length adiponectin in pregnant mice down-regulates placental amino acid transporter activity and expression and decreases fetal growth. J Physiol. 2012;590(6):1495–1509. 22289908 10.1113/jphysiol.2011.226399 PMC3382336

[27] Rosario FJ, Nathanielsz PW, Powell TL, Jansson T. Maternal folate deficiency causes inhibition of mTOR signaling, down-regulation of placental amino acid transporters and fetal growth restriction in mice. Sci Rep. 2017;7(1):3982. 28638048 10.1038/s41598-017-03888-2 PMC5479823

[28] Ozmen A, Unek G, Kipmen-Korgun D, Cetinkaya B, Avcil Z, Korgun ET. Glucocorticoid exposure altered angiogenic factor expression via Akt/mTOR pathway in rat placenta. Ann Anat. 2015;198:34–40. 25479925 10.1016/j.aanat.2014.10.007

[29] Kimball R, Wayment M, Merrill D, Wahlquist T, Reynolds PR, Arroyo JA. Hypoxia reduces placental mTOR activation in a hypoxia-induced model of intrauterine growth restriction (IUGR). Physiol Rep. 2015;3(12):e12651. 26660559 10.14814/phy2.12651 PMC4760431

[30] Mejia C, Lewis J, Jordan C et al. Decreased activation of placental mTOR family members is associated with the induction of intrauterine growth restriction by secondhand smoke in the mouse. Cell Tissue Res. 2017;367(2):387–395. 27613305 10.1007/s00441-016-2496-5

[31] Cao X, Hua X, Wang X, Chen L. Exposure of pregnant mice to triclosan impairs placental development and nutrient transport. Sci Rep. 2017;7:44803. 28322267 10.1038/srep44803 PMC5359620

[32] Dong J, Xu Q, Qian C et al. Fetal growth restriction exhibits various mTOR signaling in different regions of mouse placentas with altered lipid metabolism. Cell Biol Toxicol. 2024;40(1):15. 38451382 10.1007/s10565-024-09855-8 PMC10920423

[33] Beetch M, Akhaphong B, Wong A et al. Impact of placental mTOR deficiency on peripheral insulin signaling in adult mice offspring. J Mol Endocrinol. 2023;71(4):230035. 10.1530/JME-23-0035. PMC10620464 37855320

[34] Akhaphong B, Baumann DC, Beetch M et al. Placental mTOR complex 1 regulates fetal programming of obesity and insulin resistance in mice. JCI Insight. 2021;6(13):e149271. 10.1172/jci.insight.149271. 34032632 PMC8410096

[35] Dai Y, Sang XB, Bai WP. N-acetylcysteine and hydroxychloroquine ameliorate ADMA-induced fetal growth restriction in mice via regulating oxidative stress and autophagy. Rep Sci. 2024;31(3):779–790. 10.1007/s43032-023-01380-z 37845590

[36] Tanaka K, Tanaka H, Tachibana R et al. Tadalafil treatment of mice with fetal growth restriction and preeclampsia improves placental mTOR signaling. Int J Mol Sci. 2022;23(3):1474. 35163395 10.3390/ijms23031474 PMC8835936

[37] Yung HWa, Hemberger M, Watson ED et al. Endoplasmic reticulum stress disrupts placental morphogenesis: implications for human intrauterine growth restriction. J Pathol. 2012;228(4):554–564. 22733590 10.1002/path.4068 PMC3532660

[38] Wali JA, De Boo HA, Derraik JGB et al. Weekly intra-amniotic IGF-1 treatment increases growth of growth-restricted ovine fetuses and up-regulates placental amino acid transporters. PLoS One. 2012;7(5):e37899. 22629469 10.1371/journal.pone.0037899 PMC3358268

[39] Aye IL, Rosario FJ, Powell TL, Jansson T. Adiponectin supplementation in pregnant mice prevents the adverse effects of maternal obesity on placental function and fetal growth. Proc Natl Acad Sci USA. 2015;112(41):12858–12863. 26417088 10.1073/pnas.1515484112 PMC4611638

[40] Capobianco E, Fornes D, Roberti SL, Powell TL, Jansson T, Jawerbaum A. Supplementation with polyunsaturated fatty acids in pregnant rats with mild diabetes normalizes placental PPARgamma and mTOR signaling in female offspring developing gestational diabetes. J Nutr Biochem. 2018;53:39–47. 29190548 10.1016/j.jnutbio.2017.10.006

[41] Sati L, Soygur B, Celik-Ozenci C. Expression of mammalian target of rapamycin and downstream targets in normal and gestational diabetic human term placenta. Reprod Sci. 2016;23(3):324–332. 26335179 10.1177/1933719115602765

[42] Gaccioli F, White V, Capobianco E, Powell TL, Jawerbaum A, Jansson T. Maternal overweight induced by a diet with high content of saturated fat activates placental mTOR and eIF2alpha signaling and increases fetal growth in rats. Biol Reprod. 2013;89(4):96. 24006279 10.1095/biolreprod.113.109702

[43] Shang M, Wen Z. Increased placental IGF-1/mTOR activity in macrosomia born to women with gestational diabetes. Diabetes Res Clin Pract. 2018;146:211–219. 30389621 10.1016/j.diabres.2018.10.017

[44] Kanai Y, Segawa H, Miyamoto K-I, Uchino H, Takeda E, Endou H. Expression cloning and characterization of a transporter for large neutral amino acids activated by the heavy chain of 4F2 antigen (CD98). J Biol Chem. 1998;273(37):23629–23632. 9726963 10.1074/jbc.273.37.23629

[45] Gagné L M., Morin N, Lavoie N et al. Tyrosine phosphorylation of DEPTOR functions as a molecular switch to activate mTOR signaling. J Biol Chem. 2021;297(5):101291. 34634301 10.1016/j.jbc.2021.101291 PMC8551655

[46] Jansson T, Castillo-Castrejon M, Gupta MB, Powell TL, Rosario FJ. Down-regulation of placental Cdc42 and Rac1 links mTORC2 inhibition to decreased trophoblast amino acid transport in human intrauterine growth restriction. Clin Sci (Colch). 2020;134(1):53–70. 31825077 10.1042/CS20190794 PMC8812325

[47] Rosario FJ, Dimasuay KG, Kanai Y, Powell TL, Jansson T. Regulation of amino acid transporter trafficking by mTORC1 in primary human trophoblast cells is mediated by the ubiquitin ligase Nedd4-2. Clin Sci (Colch). 2016;130(7):499–512. 26608079 10.1042/CS20150554 PMC5681479

[48] Rosario FJ, Barentsen K, Powell TL et al. Trophoblast-specific overexpression of the LAT1 increases transplacental transport of essential amino acids and fetal growth in mice. PNAS Nexus. 2024;3(6):pgae207. 38894879 10.1093/pnasnexus/pgae207 PMC11184900

[49] Vaughan OR, Maksym K, Silva E et al. Placenta-specific Slc38a2/SNAT2 knockdown causes fetal growth restriction in mice. Clin Sci (Colch). 2021;135(17):2049–2066. 34406367 10.1042/CS20210575 PMC8410983

[50] Dumolt JH, Rosario FJ, Barentsen K, Urschitz J, Powell TL, Jansson T. Trophoblast-specific overexpression of adiponectin receptor 2 causes fetal growth restriction in pregnant mice. FASEB J. 2024;38(19):e70100. 39387608 10.1096/fj.202302143R PMC11508969

[51] Moffat J, Grueneberg DA, Yang X et al. A lentiviral RNAi library for human and mouse genes applied to an arrayed viral high-content screen. Cell. 2006;124(6):1283–1298. 16564017 10.1016/j.cell.2006.01.040

[52] Harrison SE, Sozen B, Zernicka-Goetz M. In vitro generation of mouse polarized embryo-like structures from embryonic and trophoblast stem cells. Nat Protoc. 2018;13(7):1586–1602. 29988106 10.1038/s41596-018-0005-x

[53] Byers SL, Wiles MV, Dunn SL, Taft RA. Mouse estrous cycle identification tool and images. PLoS One. 2012;7(4):e35538. 22514749 10.1371/journal.pone.0035538 PMC3325956

[54] Kusinski LC, Jones CJ, Baker PN, Sibley CP, Glazier JD. Isolation of plasma membrane vesicles from mouse placenta at term and measurement of system A and system beta amino acid transporter activity. Placenta. 2010;31(1):53–59. 19954844 10.1016/j.placenta.2009.11.006 PMC2877806

[55] Rosario FJ, Kanai Y, Powell TL, Jansson T. Mammalian target of rapamycin signalling modulates amino acid uptake by regulating transporter cell surface abundance in primary human trophoblast cells. J Physiol. 2013;591(3):609–625. 23165769 10.1113/jphysiol.2012.238014 PMC3577540

[56] Gaziano EP . Antenatal ultrasound and fetal Doppler. Diagnosis and outcome in intrauterine growth retardation. Clin Perinatol. 1995;22(1):111–140. 7781247

[57] Arbuckle TE, Wilkins R, Sherman GJ. Birth weight percentiles by gestational age in Canada. Obstet Gynecol. 1993;81(1):39–48. 8416459

[58] Gelbaya TA, Nardo LG. Customised fetal growth chart: a systematic review. J Obstet Gynaecol. 2005;25(5):445–450. 16183577 10.1080/01443610500160444

[59] Barkehall‐Thomas A, Wilson C, Baker L, Bhuinneain MNi, Wallace EM. Uterine artery doppler velocimetry for the detection of adverse obstetric outcomes in patients with elevated mid-trimester beta-human chorionic gonadotrophin. Acta Obstet Gynecol Scand. 2005;84(8):743–747. 16026398 10.1111/j.0001-6349.2005.00798.x

[60] Alessi DR, Pearce LR, García-Martínez JM. New insights into mTOR signaling: mTORC2 and beyond. Sci Signal. 2009;2(67):pe27. 19383978 10.1126/scisignal.267pe27

[61] Zoncu R, Efeyan A, Sabatini DM. mTOR: from growth signal integration to cancer, diabetes and ageing. Nat Rev Mol Cell Biol. 2011;12(1):21–35. 21157483 10.1038/nrm3025 PMC3390257

[62] Rosario FJ, Kanai Y, Powell TL, Jansson T. Increased placental nutrient transport in a novel mouse model of maternal obesity with fetal overgrowth. Obesity (Silver Spring). 2015;23:(8):1663–1670. 26193061 10.1002/oby.21165 PMC4509489

[63] Capobianco E, Fornes D, Linenberg I, Powell TL, Jansson T, Jawerbaum A. A novel rat model of gestational diabetes induced by intrauterine programming is associated with alterations in placental signaling and fetal overgrowth. Mol Cell Endocrinol. 2016;422:221–232. 26747729 10.1016/j.mce.2015.12.020

[64] Li S, Lu T, Lin Z et al. Supplementation with probiotics co-cultivation improves the reproductive performance in a sow-piglet model by mother-infant microbiota transmission and placental mTOR signaling. World J Microbiol Biotechnol. 2025;41(1):13. 10.1007/s11274-024-04222-5 39704872

[65] Shao X, Cao G, Chen D et al. Placental trophoblast syncytialization potentiates macropinocytosis via mTOR signaling to adapt to reduced amino acid supply. Proc Natl Acad Sci USA. 2021;118:(3):e2017092118. 10.1073/pnas.2017092118. 33402432 PMC7826386

[66] Fahlbusch FB, Hartner A, Menendez-Castro C et al. The placental mTOR-pathway: correlation with early growth trajectories following intrauterine growth restriction?. J Dev Orig Health Dis. 2015;6(4):317–326. 25989725 10.1017/S2040174415001154

[67] Oh S-Y, Hwang JR, Lee Y et al. Isolation of basal membrane proteins from BeWo cells and their expression in placentas from fetal growth-restricted pregnancies. Placenta. 2016;39:24–32. 26992671 10.1016/j.placenta.2016.01.001

[68] Gao K, Jiang Z, Lin Y et al. Dietary L-arginine supplementation enhances placental growth and reproductive performance in sows. Amino Acids. 2012;42(6):2207–2214. 21691753 10.1007/s00726-011-0960-9

[69] Greene JM, Dunaway CW, Bowers SD, Rude BJ, Feugang JM, Ryan PL. Dietary L-arginine supplementation during gestation in mice enhances reproductive performance and Vegfr2 transcription activity in the fetoplacental unit. J Nutr. 2012;142(3):456–460. 22279135 10.3945/jn.111.154823

[70] Liu T, Zuo B, Wang W, Wang S, Wang J. Dietary supplementation of leucine in premating diet improves the within-litter birth weight uniformity, antioxidative capability, and immune function of primiparous SD rats. Biomed Res Int. 2018;2018:1523147. 29850484 10.1155/2018/1523147 PMC5932505

[71] Cui C, Wu C, Wang J et al. Leucine supplementation during late gestation globally alters placental metabolism and nutrient transport via modulation of the PI3K/AKT/mTOR signaling pathway in sows. Food Funct. 2022;13(4):2083–2097. 35107470 10.1039/d1fo04082k

[72] Desforges M, Parsons L, Westwood M, Sibley CP, Greenwood SL. Taurine transport in human placental trophoblast is important for regulation of cell differentiation and survival. Cell Death Dis. 2013;4(3):e559. 23519128 10.1038/cddis.2013.81 PMC3618382

[73] Li S, Ye X, Wen X et al. Arginine and its metabolites stimulate proliferation, differentiation, and physiological function of porcine trophoblast cells through beta-catenin and mTOR pathways. BMC Vet Res. 2024;20(1):167. 38689278 10.1186/s12917-024-04023-w PMC11062007

[74] Shen SF, Hua CH. Effect of L-arginine on the expression of bcl-2 and bax in the placenta of fetal growth restriction. J Matern Fetal Neonatal Med. 2011;24(6):822–826. 21067292 10.3109/14767058.2010.531315

[75] Gouvêa VN, Smithyman MM, Hentz F, Bagheri N, Batistel F. Methionine supply during mid-gestation modulates the bovine placental mTOR pathway, nutrient transporters, and offspring birth weight in a sex-specific manner. J Anim Sci. 2024;102:skae305. 10.1093/jas/skae305. 39390894 PMC11537801

[76] Batistel F, Alharthi ASm, Wang L et al. Placentome nutrient transporters and mammalian target of rapamycin signaling proteins are altered by the methionine supply during late gestation in dairy cows and are associated with newborn birth weight. J Nutr. 2017;147(9):1640–1647. 28768834 10.3945/jn.117.251876

[77] Rosario FJ, Urschitz​​​​​​ J, Razavy H, Elston M, Theresa PL, Jansson T. PiggyBac transposase mediated inducible trophoblast-specific knockdown of mechanistic target of Rapamycin in mice decreases placental nutrient transport and inhibits fetal growth [published online ahead of print February 22, 2024]. 2024. Biorxiv. 10.1101/2024.02.20.581180.

[78] Viola I, Accornero P, Manenti I, Miretti S, Baratta M, Toschi P. mTOR is an essential gate in adapting the functional response of ovine trophoblast cells under stress-inducing environments. Placenta. 2024;158:14–22. 39341011 10.1016/j.placenta.2024.09.011

[79] Barroso E, Díaz M, Reguera AC et al. CHOP upregulation and dysregulation of the mature form of the SNAT2 amino acid transporter in the placentas from small for gestational age newborns. Cell Commun Signal. 2023;21(1):326. 37957724 10.1186/s12964-023-01352-5 PMC10644500

[80] Vaughan OR, Rosario FJ, Chan J et al. Maternal obesity causes fetal cardiac hypertrophy and alters adult offspring myocardial metabolism in mice. J Physiol. 2022;600(13):3169–3191. 35545608 10.1113/JP282462

[81] Dumolt J, Powell TL, Jansson T, Rosario FJ. Normalization of maternal adiponectin in obese pregnant mice prevents programming of impaired glucose metabolism in adult offspring. FASEB J. 2022;36(7):e22383. 35670755 10.1096/fj.202200326R PMC9202510

