# Supplementary figures and images for: Lentivirus-Mediated Trophoblast-Specific Deptor Knockdown Increases Transplacental System A and System L Amino Acid Transport and Fetal Growth in Mice
Source: Function (Oxf). 2025 Mar 25;6(2):zqaf018. doi: 10.1093/function/zqaf018 (PMC11992690; doi:10.1093/function/zqaf018)

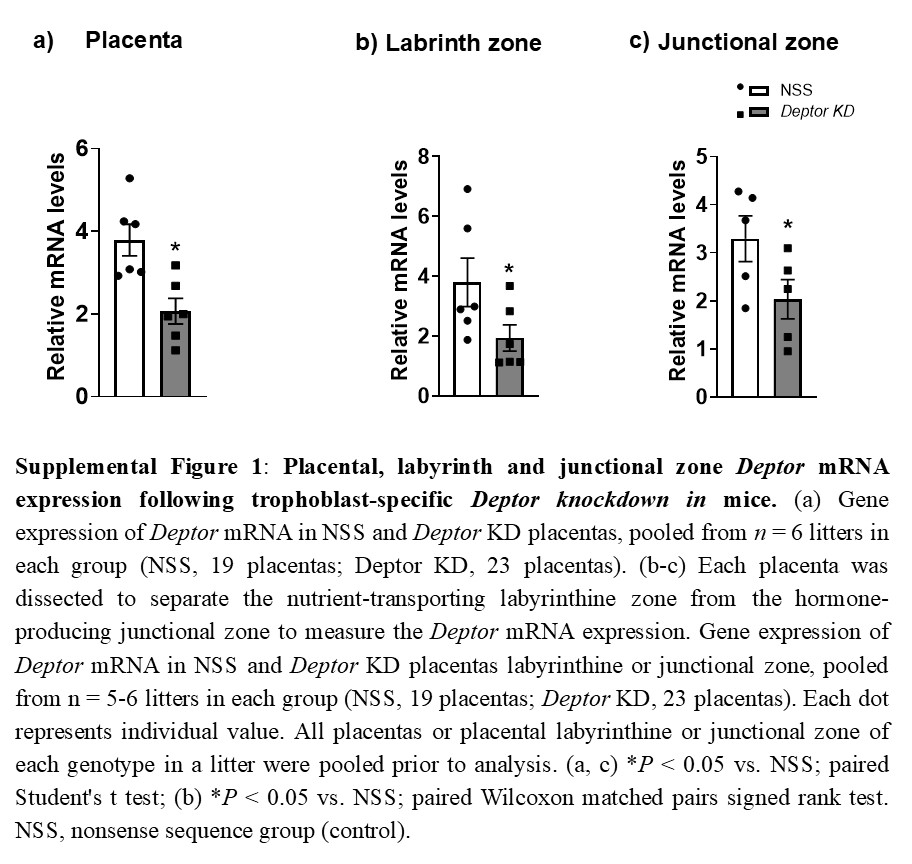

Supplement: zqaf018_Supplemental_Files [file zqaf018_supplemental_files.zip › Supplemental Figure 1.jpg]

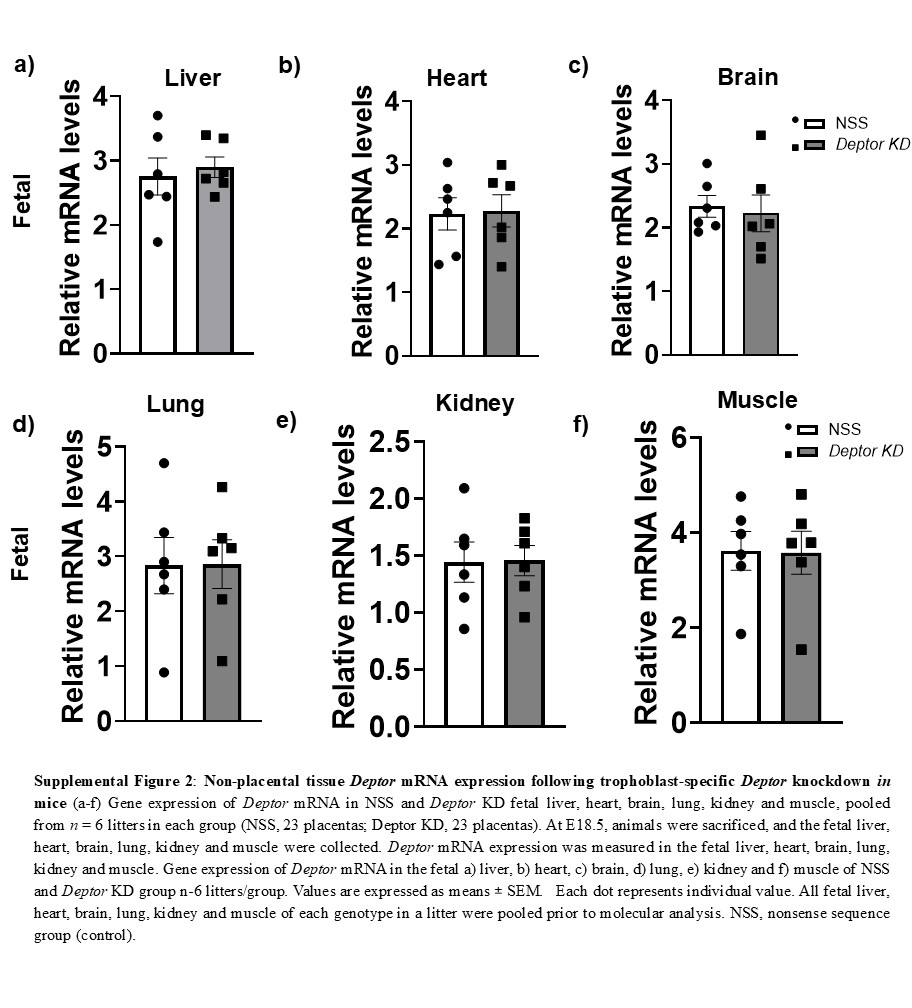

Supplement: zqaf018_Supplemental_Files [file zqaf018_supplemental_files.zip › Supplemental Figure 2.jpg]

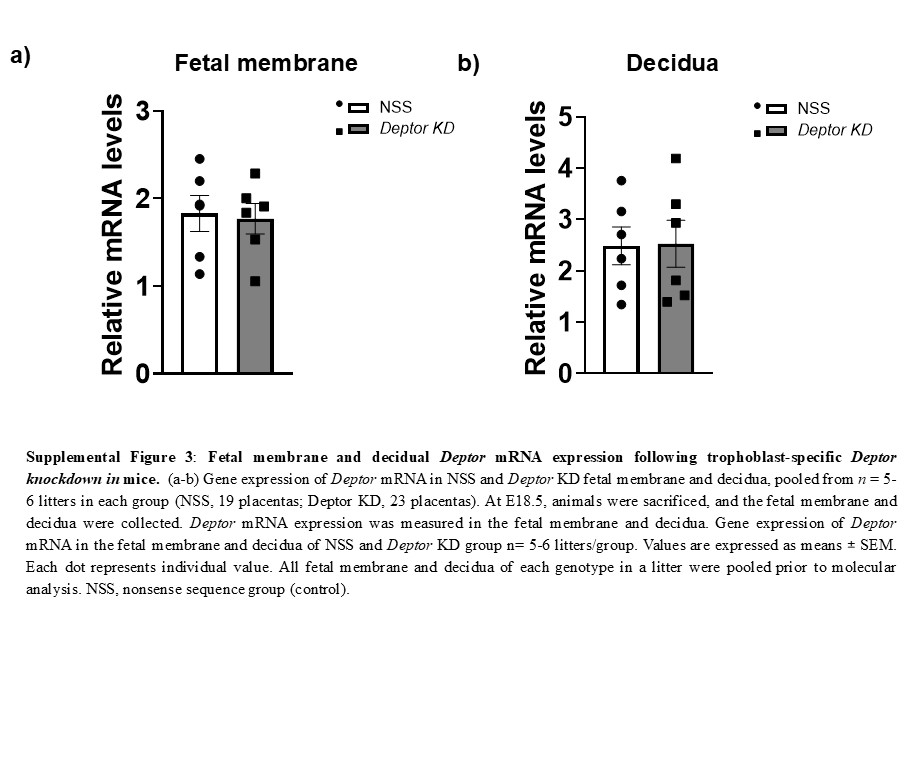

Supplement: zqaf018_Supplemental_Files [file zqaf018_supplemental_files.zip › Supplemental Figure 3.jpg]

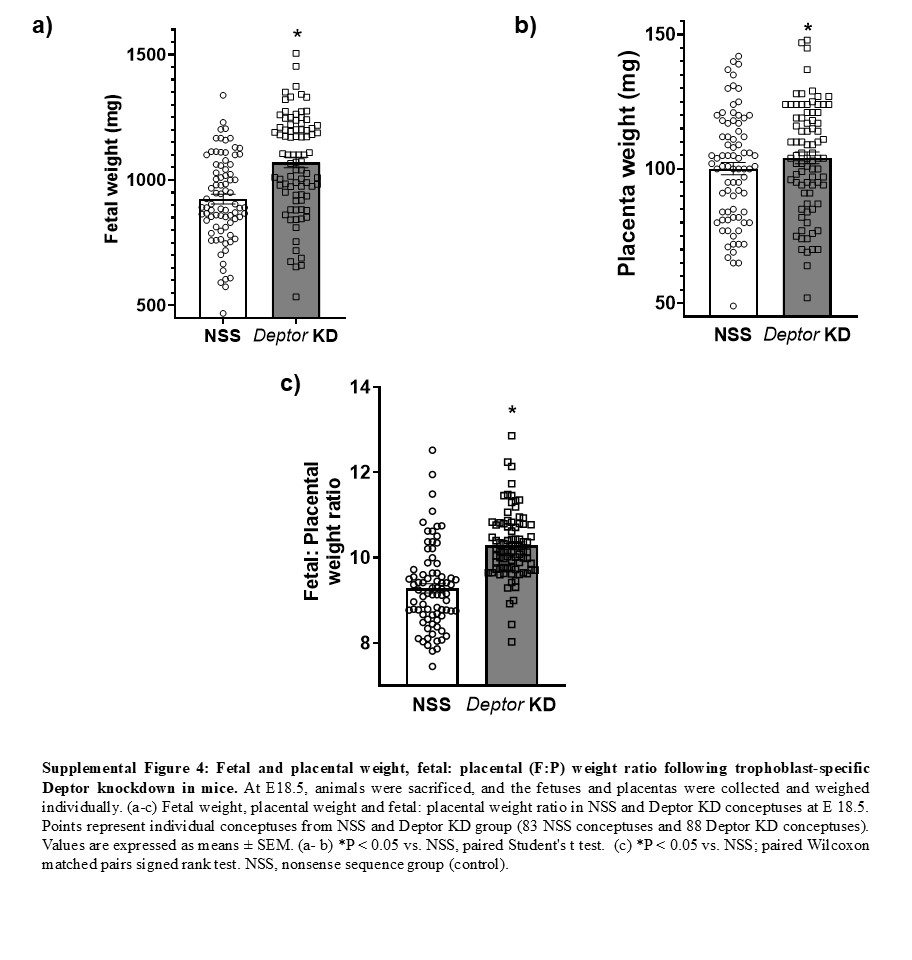

Supplement: zqaf018_Supplemental_Files [file zqaf018_supplemental_files.zip › Supplemental Figure 4.jpg]

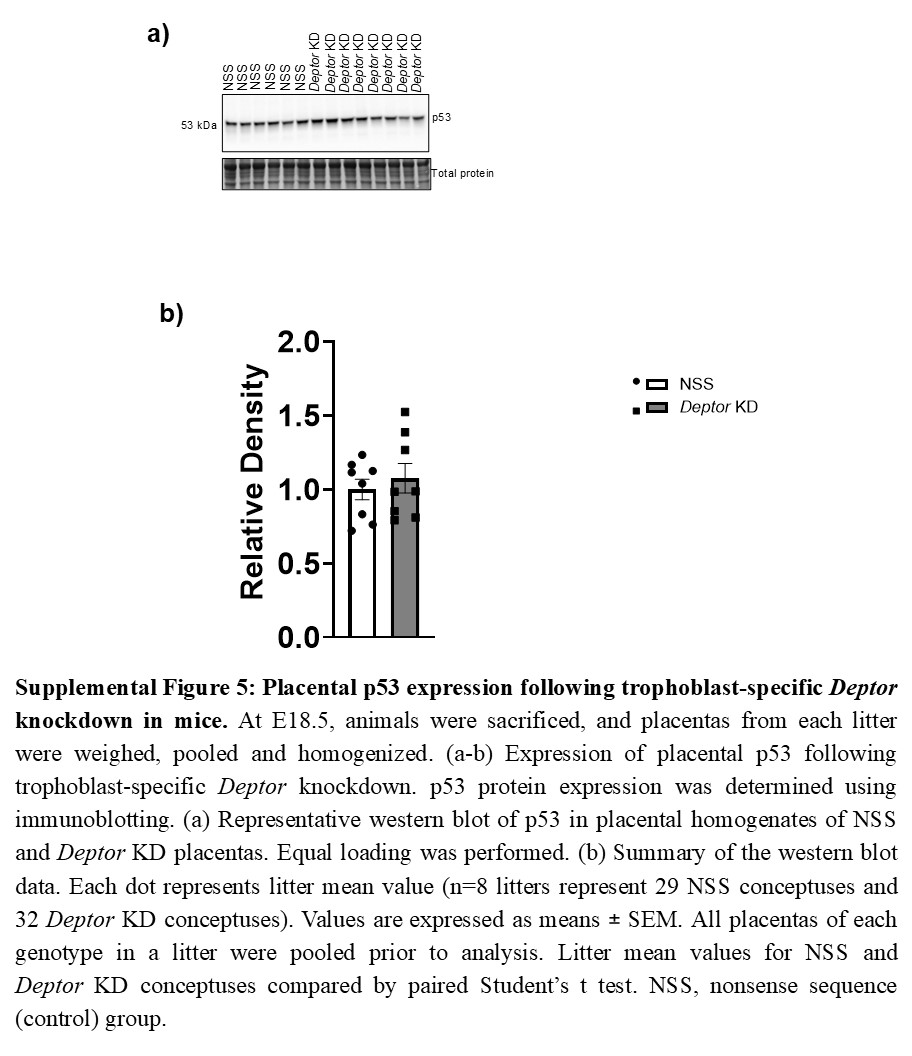

Supplement: zqaf018_Supplemental_Files [file zqaf018_supplemental_files.zip › Supplemental Figure 5.jpg]
